# CD47 Expression in Natural Killer Cells Regulates Homeostasis and Modulates Immune Response to Lymphocytic Choriomeningitis Virus

**DOI:** 10.3389/fimmu.2018.02985

**Published:** 2018-12-20

**Authors:** Pulak Ranjan Nath, Arunakumar Gangaplara, Dipasmita Pal-Nath, Ajeet Mandal, Dragan Maric, John M. Sipes, Maggie Cam, Ethan M. Shevach, David D. Roberts

**Affiliations:** ^1^Laboratory of Pathology, Center for Cancer Research, National Cancer Institute, National Institutes of Health, Bethesda, MD, United States; ^2^Laboratory of Immunology, National Institute of Allergy and Infectious Diseases, National Institutes of Health, Bethesda, MD, United States; ^3^CCR Collaborative Bioinformatics Resource, Office of Science and Technology Resources, National Cancer Institute and Leidos Biomedical Research, Inc., National Institutes of Health, Bethesda, MD, United States; ^4^National Institute of Neurological Disorders and Stroke, National Institutes of Health, Bethesda, MD, United States

**Keywords:** CD47, natural killer cells, antisense morpholino, mouse models, viral immunity, lymphocytic choriomeningitis virus, transcriptome analysis, RNA-seq

## Abstract

CD47 is a ubiquitous cell surface receptor that directly regulates T cell immunity by interacting with its inhibitory ligand thrombospondin-1 and limits clearance of cells by phagocytes that express its counter-receptor signal-regulatory protein-α. Murine natural killer (NK) cells express higher levels of CD47 than other lymphocytes, but the role of CD47 in regulating NK cell homeostasis and immune function remains unclear. *Cd47*^−/−^ mice exhibited depletion of NK precursors in bone marrow, consistent with the antiphagocytic function of CD47. In contrast, antisense CD47 knockdown or gene disruption resulted in a dose dependent accumulation of immature and mature NK cells in spleen. Mature *Cd47*^−/−^ NK cells exhibited increased expression of NK effector and interferon gene signatures and an increased proliferative response to interleukin-15 *in vitro*. *Cd47*^−/−^ mice showed no defect in their early response to acute Armstrong lymphocytic choriomeningitis virus (LCMV) infection but were moderately impaired in controlling chronic Clone-13 LCMV infection, which was associated with depletion of splenic NK cells and loss of effector cytokine and interferon response gene expression in *Cd47*^−/−^ NK cells. Broad CD47-dependent differences in NK activation, survival, and exhaustion pathways were observed in NK cell transcriptional signatures in LCMV infected mice. These data identify CD47 as a cell-intrinsic and systemic regulator of NK cell homeostasis and NK cell function in responding to a viral infection.

## Introduction

Natural killer (NK) cells are a distinct group of cytotoxic innate lymphoid cells that play significant roles as a first line of defense against pathogenic invasion and malignant cell transformation (1–5). These lymphocytes uniquely express inhibitory receptors including CD94 and the leukocyte immunoglobulin-like receptor family and activating receptors including NKG2D and the natural cytotoxicity receptors (6). NK killer activity is induced in the absence of signals from inhibitory receptors or when signals from activating and co-activating receptors overcome inhibitory signals. The development and education of NK cells broadly rely on the interactions of inhibitory and activating NK cell receptors with target MHC class I molecules (7–9). NK cells also regulate T cell–mediated immune response in the context of autoimmunity, transplantation and viral infection (10–12). Though the development and function of NK cells are widely studied, our understanding of the cell intrinsic factors regulating homeostasis and function of NK cells is incomplete.

CD47 is a transmembrane protein that interacts with integrins, some counter-receptor signal-regulatory protein (SIRP) family members, and the secreted thrombospondin-1 (13–16). CD47 has two established roles in the immune system. Its engagement of SIRPα on phagocytic cells and dendritic cells induces inhibitory signals that limit phagocytosis of CD47-expressing cells and antigen presentation (17). CD47-targeted therapeutics have been developed to overcome this immune checkpoint for cancer treatment (15, 18). Conversely, engagement of CD47 by thrombospondin-1 inhibits T cell receptor signaling and antigen presentation by dendritic cells (19–22). CD47 is, therefore, a checkpoint that regulates both innate and adaptive immunity. Immune responses to infection in *Cd47*^−/−^ mice support this dual role for CD47 and identified alterations in functional responses of neutrophils, macrophages, and T cells (23–28). Loss of CD47 can impair or enhance immunity to specific bacterial or fungal infections. However, its role in viral immunity is poorly understood but will be important for evaluating experimental cancer therapeutics targeting CD47.

NK cells play direct and indirect roles in anti-viral immunity (5, 11, 29, 30), but the cell-intrinsic role of CD47 in NK cells remains poorly understood. One study reported that thrombospondin-1 inhibits early NK cell proliferation and enhances late expansion, but a role for CD47 was not examined (31). CD47 as a SIRPα counter-receptor was implicated in engraftment of NK precursors in mice reconstituted with a human immune system (32). Treatment with an inhibitory CD47 antibody increased NK cell killing of head-and-neck squamous carcinoma cells *in vitro*, but the mechanism remains unclear because NK cells are not known to express SIRPα (33). Depletion of NK cells similarly attenuated the anti-tumor activity of a SIRPα blocking antibody in a syngeneic murine renal carcinoma model, but the same antibody did not inhibit NK killing of the tumor cells *in vitro*, further supporting a SIRPα-independent function of CD47 in NK cells (34). Here we report increased abundance of lineage-negative cells within the spleen of mice as a consequence of acute CD47 knockdown using an antisense morpholino or CD47 genetic deficiency. Further, studies identified these cells to be a subset of immature NK lineage. We therefore examined effects of CD47-deficiency on peripheral NK cell homeostasis, and also NK cell responses to acute and chronic LCMV infections in mice. Global transcriptome analyses of wildtype (WT) and *Cd47*^−/−^ NK cells and responses to *ex vivo* stimulation indicate that cell-intrinsic expression of CD47 regulates peripheral NK cell homeostasis and responses to exogenous immune stimuli.

## Materials and Methods

### Ethics Statement

All animal experiments were carried out in strict accordance with the recommendations for the Care and Use of Laboratory Animals of the National Institutes of Health. The protocols were approved by the NCI Animal Care and Use Committee (Protocol No: NCI/LP-012) and by the National Institute of Allergy and Infectious Diseases Animal Care and Use Committee (Protocol No: LI-5E).

### Mice

Breeding pairs of WT and B6.129S7-Cd47^tm1Fpl^/J (*Cd47*^−/−^) C57BL/6J mice were purchased from the Jackson Laboratory, CD45.1-expressing B6.SJL-Ptprca Pepcb/BoyJ strain (SJL) was obtained from Taconic Laboratory, NIAID. *Cd47*^−/−^ mice were backcrossed to minimize genetic drift and obtain *Cd47*^+/−^ mice. Heterozygous *Cd47*^+/−^ breeding pairs were set-up to obtain *Cd47*^+/+^*, Cd47*^+/−^*, and Cd47*^−/−^ littermate mice. Mice were maintained and bred under specific pathogen-free conditions under protocol NCI/LP-012. Littermate and sex-matched mice were used between 6 and 12 weeks of age for experiments, unless otherwise indicated. Injections of morpholino were done under NCI/LP-012, whereas LCMV infection of mice was done under NIAID/LI-5E protocol.

### Reagents

A translation-blocking antisense morpholino oligonucleotide complementary to CD47 (CGTCACAGGCAGGACCCACTGCCCA) was obtained from GeneTools as previously described (35). 4′,6-diamidino-2-phenylindole (DAPI) (Cat#D9542), rat serum (Cat#R9759) and rabbit serum (Cat#R9133) were purchased from Sigma-Aldrich. Aqua live/dead was from ThermoFisher Scientific (Cat#L3495), UV Zombie from BioLegend (Cat#423107), and Ammonium-Chloride-Potassium (ACK) lysis buffer was from Lonza (Cat#10-548E).

### Tissue Processing

For isolation of bone marrow hematopoietic cells, femurs from hind legs of mice were separated, both ends were perfused with a 22G needle, and cells within the bone were flushed out in complete RPMI. Red blood cells (RBCs) were lysed using ACK buffer, and cells were resuspended in FACS buffer. Liver and lungs were cut into small pieces and enzymatically dissociated with Collagenase/Dispase (Roche, Cat# 269638, final concentration 1 mg/ml) and DNase 1 (Sigma, Cat# D4527, final concentration 100 μg/ml). Cells were then filtered through a 70 μm strainer. Thymus, spleen and mLN were isolated and mechanically disrupted in complete RPMI. RBCs were lysed with ACK buffer, and single cell suspensions were filtered through a 70 μm strainer.

### Pan T/iNK Cell Isolation

A total peripheral T cell population that includes iNK cells was isolated from mouse spleens by negative selection using Pan T cell Isolation Kit II (MACS, Miltenyi Biotec) to deplete cells expressing CD11b, CD11c, CD19, CD45R, CD49b, CD105, MHC class II, and Ter-119. CD4^+^, CD8^+^, CD3^+^CD4^−^CD8^−^(DN), and CD3^−^DN cells from the isolated Pan T/iNK cells were further sorted using FACSAria II (BD Biosciences). Sorted CD3^−^ DN cells were cultured in complete RPMI with IL-2 (60 IU/ml).

### ImageStream Analysis

Pan T/iNK cells from the spleens of WT and *Cd47*^−/−^ littermate mice were isolated, and cells were immunolabeled using a combination of spectrally compatible fluorophore-conjugated antibodies against CD3, CD4, CD8, and CD45. The cells were then fixed (IC Fixation Buffer, eBioscience) and nuclei counterstained with 1 μg/ml DAPI to facilitate visualization and counting of intact cells. The expression of the above combinations of markers on individual cells was quantified using an ImageStream^X^ imaging flow cytometer (Amnis/EMD Millipore) equipped with a 12-channel, dual camera fluorescence detection system sensitive to fluorophores emitting from violet to near-infra red-light spectrum and the following lasers for excitation: 405, 488, and 658 nm. Cell imagery and phenotyping data analysis was processed and counted using IDEAS image analysis software (Amnis) per the manufacturer's recommendations.

### Cell Proliferation Analysis by Flow Cytometry

Sorted cells were pulsed either with 5 μM carboxyfluorescein diacetate succinimidyl ester (CFSE) or 5 μM CellTrace Violet (CTV) for 30 min at room temperature protected from light. Cells were then washed and cultured in complete RPMI with the indicated stimuli. Cell proliferation was followed for 2 or 7 days. Cells were acquired using a BD LSR Fortessa SORP Flow Cytometer with 355-nm excitation and a 450/50-nm bandpass emission filter. The discrete peaks in the histograms represent successive generations of live NK cells. An overlay of the unstimulated parent generation is indicated as the brightest peak on the far-right side of the histograms.

### MTS Proliferation Assay and ELISA

Unlabeled NK cells were isolated by depletion of non-target cells using the NK Cell Isolation Kit (Miltenyi Biotech, cat# 130-115-818) from spleens of WT and littermate *Cd47*^−/−^ mice. Isolated NK cells were cultured in 24 well flat bottom cell culture plates on complete RPMI and/or with 40 ng/mL IL15. The cultures were inoculated with 10^6^ and 10^7^ cells /mL for cell proliferation assay (MTS) and ELISA, respectively. Cell proliferation was estimated for 24 and 48 h using CellTiter 96® AQueous Non-Radioactive Cell Proliferation Assay (MTS) (Promega, cat# G5421). Levels of the secreted cytokine IFNγ and granzyme B (GzmB) in the NK cell culture media were quantified using ELISA kits: RayBio® Mouse IFN-gamma B Elisa Kit (cat# ELM-IFNγ) and RayBio® Mouse Granzyme B Elisa Kit (cat# ELM-GranzymeB), respectively.

### Bone Marrow Chimera

The WT and *Cd47*^−/−^ mice express the CD45.2 isoform. Therefore, CD45.1 mice were used to evaluate bone marrow chimera reconstitution. Total body irradiation was performed on 8 weeks old immunocompetent CD45.1 mice irradiated with two separate doses of 550 Rads within an interval of 3 h. The next day, bone marrow derived cells from femurs of donor CD45.2^+^ WT and *Cd47*^−/−^ mice and recipient CD45.1^+^ mice were subjected to Thy1.2^+^ (CD90.2) and NK cell depletion using anti-CD90.2 and anti-NK1.1 antibodies. Donor and recipient bone marrow derived cells were mixed at 1:1 ratio and injected to the irradiated recipient CD45.1 mice (2 × 10^6^ cells/mouse, i.v). To avoid any systemic bacterial infection, the recipient mice were put on Trimethoprim Sulfa-treated water for 3 weeks after irradiation. Eight weeks later, peripheral blood from these mice was collected and analyzed by flow cytometer. The degree of reconstitution was evaluated based on the differential expression of CD45 isoforms.

### LCMV Infection and Plaque Assay

LCMV Armstrong and Cl-13 viruses (Shevach Laboratory) were propagated in baby hamster kidney-21 fibroblast cells [American Type Culture Collection (ATCC), Manassas, VA, United States]. Viral titers were determined by plaque assay using Vero African-green-monkey kidney cells (ATCC). Viral stocks were frozen at −80°C until used. Mice were infected with the diluted virus in 1x sterile phosphate buffer saline (PBS) (Armstrong virus, 2 × 10^5^ plaque forming unit (pfu)/mouse, i.p., or Cl-13 virus, 2 × 10^6^ pfu/mouse, i.v.). LCMV titers in sera were determined by plaque assay using Vero cells as described (36).

### RNA Extraction, Quantitative Real-Time PCR and Primer Sequences

RNA was purified from the indicated cell types using the RNeasy Microkit (Qiagen) or TRIzol following manufacturer's instructions. RNA was reverse transcribed to cDNA using Thermo Scientific Maxima First Strand cDNA Synthesis Kit for RT-qPCR. Quantitative real-time PCR was performed with SYBR Green using primers for specific genes (Table S3), and analyzed on CFX96 Real-time System (Bio Rad). Relative transcript abundance was determined by using the ΔΔ*C*_*t*_ or Δ*C*_*t*_ method after normalization with β*-Actin* and *Gapdh*. All samples were run in triplicate. Error bars represent S.E.M.

### Flow Cytometry and Cell Sorting

Single cell suspensions from organs and tissues were prepared as described above. Cell preparations were stained with optimized antibody dilutions. Antibodies used in the lineage cocktail (Lin) include, but not limited to, antibody against B220 (RA3-6B2), CD19 (eBioD3), Gr1 (RB6-8C5), CD11c (N418), and Ter119 (TER-119). Additional antibodies used included those targeting antibody molecules CD45.2 (104), CD4 (RM4-5), CD8 (53-6.7), CD3 (145-2C11), NK1.1 (PK136), NKp46 (29A1.4), CD122 (TM-b1), CD127 (A7R34), CD49b (DX5), CD11b (ICRF44), CD11c (HL3), CD69 (H1.2F3), CD44 (IM7), CD62L (MEL-14), KLRG1 (2F1/KLRG1), TCR-β (H57-597), PD1 (29F.1A12), CD47 (miap301), and intracellular molecules TNF-α (MP6-XT22), Ki-67 (SolA15, B56), Eomes (Dan11mag), T-bet (eBio4B10), IFN-γ (XMG1.2) and Granzyme B (NGZB, GB11). Antibodies were directly conjugated to Brilliant Ultraviolet (BUV)395, Brilliant Violet (BV)786, BV711, BV650, BV605, Pacific Blue (PB), fluorescein isothiocyanate (FITC), phycoerythrin (PE), PE-Cy5.5, PE-Texas Red, peridinin-chlorophyll-protein complex (PerCP)-Cy5.5, PE-Cy7, allophycocyanin (APC), APC-Alexa 700, or biotin. Biotinylated antibodies were revealed with Streptavidin APCeFluor780. All antibodies were purchased from either eBioscience/Biolegend/BD Pharmingen. Cells were resuspended in FACS buffer (1% BSA+0.01% NaN_3_ in PBS1x, filtered) and incubated with rat plus rabbit serum followed by incubation with antibody cocktail against surface molecules. For intracellular staining, cells were fixed (IC Fixation Buffer, eBioscience) and permeabilized (Permeabilization Buffer 10x, eBioscience) and incubated with antibodies against intracellular molecules.

Cell sorting was performed on a FACSAria II (BD Biosciences), and flow cytometric analysis was performed on a LSR Fortessa SORP (BD Biosciences). Dead cells were excluded through 4,6 diamidino-2- phenylindole (DAPI) uptake or Aqua live/dead staining. Doublets were excluded through forward scatter–height by forward scatter–width and side scatter–height by side scatter–width parameters. Data were analyzed using FlowJo (Tree Star).

Flow cytometric analysis of NK cell precursors (NKP) is defined by expression of the IL-15 receptor β chain (CD122), and lack of common lineage markers, including the NK cell markers NK1.1 and DX5 (CD49b) (37). NKP (Lin^−^CD127^+/−^CD122^+^) further differentiate into immature NK (iNK) cells, which lose expression of CD127 (IL-7Rα) and acquire expression of NK1.1 but do not yet express DX5 (37). As iNK (Lin^−^CD127^−^CD122^+^NK1.1^+^DX5^−^) cells lose expression of CD122 and gain DX5, they also gain functional competence in cytotoxicity and production of interferon (IFN)-γ, and become mature NK (mNK, Lin^−^CD127^−^CD122^−^NK1.1^+^DX5^+^) cells (38, 39).

### RNA-seq Library Construction and Illumina Sequencing

Lin^−^NK1.1^+^NKp46^+^ cells were sorted from spleens of uninfected and LCMV infected mice. Total RNA was isolated from sorted cells using TRIzol (Ambion part of Life Technologies) following the manufacturer's protocols. During RNA isolation, DNase treatment was additionally performed using the RNase-free DNase set (Qiagen). RNA quality was checked using an Agilent 2100 Expert bioanalyzer (Agilent Technologies). RNA quality was reported as a score from 1 to 10, and samples falling below the threshold of 8.0 were excluded from the study. cDNA library preparation involves the removal of ribosomal RNA (rRNA) using biotinylated, target-specific oligos combined with Ribo-Zero rRNA removal beads. The RNA is fragmented into small pieces and the cleaved RNA fragments are copied into first strand cDNA using reverse transcriptase and random primers, followed by second strand cDNA synthesis using DNA Polymerase I and RNase H. The resulting double-strand cDNA is used as the input to a standard Illumina library prep with end-repair, adapter ligation and PCR amplification being performed to get a library that is ready to go to the sequencer. The final purified product is then quantified by qPCR before cluster generation and sequencing. Paired-end sequencing (2 × 150 bp) of stranded total RNA libraries was performed on one HiSeq run with Illumina HiSeq3000/4000 chemistry pair end sequencing. The samples have 99–148 million pass filter reads with a base call quality of above 92% of bases with Q30 and above.

The HiSeq Real Time Analysis software (RTA 1.18) was used for processing image files, the Illumina bcl2fastq1.8.4 was used to demultiplex and convert binary base calls and qualities to fastq format. The sequencing reads were trimmed to remove adapters and low-quality bases using Trimmomatic (version 0.30), and the trimmed reads were mapped to mouse reference genome (GRCm38/mm10) and Gencode annotation M9 using STAR (version 2.5) with two-pass alignment option. RSEM (version 1.2.22) was used for transcript quantification.

### RNA-seq Data Processing and Analysis

The reference genome and annotation from GENECODE Mouse (mm10) and Lymphocytic choriomeningitis virus (NC_004291.1) were used to merge and create genome index by using STAR version 2.5.2b. Further for the downstream analyses, an in-house pipeline (Pipeliner: https://github.com/CCBR/Pipeliner) was used as follows. To remove the adapter sequences cutadapt/1.14 was used, and followed by fastQ Screen v0.9.3 to screen the library for its composition. For quality control the tool fastqc/0.11.5 was used, and the spliced transcripts alignment to a reference genome and counting the number of reads per gene was performed by STAR/2.5.2b. The mapped reads in the genomic features were counted by subread/1.5.1, and the distribution of these reads was calculated by rseqc/2.6.4 along with the estimation of strandness of the reads. The library complexity was estimated by preseq/2.0.3. To mark duplicate reads and estimate the gene coverage the program picard/1.119, and for the overall statistics of input datasets samtools/1.5 was used. For differential expression analysis, the low abundant gene threshold was set to include genes with ≥0.5 counts per million (CPM) in at least ≥4 samples. In order to generate the gene lists based on different contrast the R Bioconductor package DESeq2 was used. PCA analysis was performed using Partek Genomics Suite (v 6.5) and Gene Set Enrichment Analysis (GSEA) performed using software provided by the Broad Institute (40).

### Statistical Analysis

Graphs were generated and statistical analysis on groups with limited variance was performed using GraphPad Prism 7 (Version 7.01). Comparison between two groups was done via unpaired two-tailed Student's *t*-test. Differences with a *p* < 0.05 were considered significant.

## Results

### CD47 Deficiency Increases NK-Lineage Cell Populations in Peripheral Lymphoid Organs

**Figure 1:**
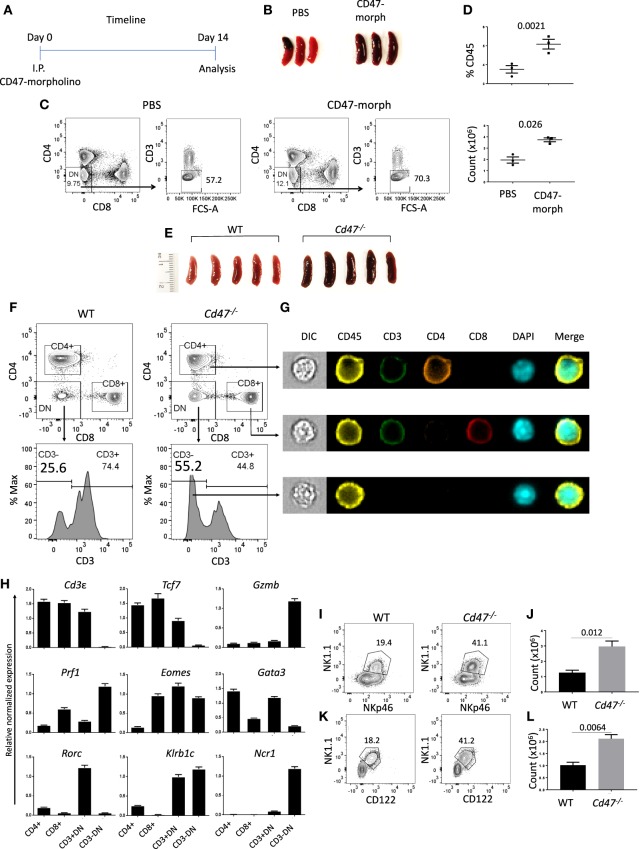
CD47 deficiency increases immature NK-lineage cells in spleen. **(A)** Time line showing C57BL/6 mice received i.p. injections of 750 μl PBS or CD47-morpholino (10 μmol/L in PBS) and were analyzed 2 weeks later. **(B)** Morphology of spleens at day 14 of treated and control mice. **(C,D)** Splenocytes were stained for lineage (Lin: B220, CD19, CD11b, CD11c, CD49b, CD105, MHC-II, and Ter119), CD3, CD4, and CD8 and acquired in a flow cytometer. DAPI was used to discriminate live/dead cells. Representative contour plots (values indicate percentage of parent population), frequency and counts of live Lin^−^CD3^−^CD4^−^CD8^−^ cells in PBS vs. CD47-morpholino treated mice are shown, *n* = 3. **(E)** Morphology of spleens was depicted from WT and *Cd47*^−/−^ littermate mice. **(F–G)** Splenocytes were enriched for lineage-negative cells and stained for CD3, CD4, and CD8. FACS plots and ImageStream analysis show staining for live Lin^−^-CD4^+^, -CD8^+^, -CD3^+^CD4^−^CD8^−^ (-CD3^+^DN) and -CD3^−^CD4^−^CD8^−^ (-CD3^−^DN) cells in WT and *Cd47*^−/−^ spleens. **(H)** RT-qPCR analysis of Lin^−^-CD4^+^, -CD8^+^, -CD3^+^DN and -CD3^−^DN populations sorted from spleens of *Cd47*^−/−^ mice was performed, β*-Actin* and *Gapdh* used as reference genes and relative normalized expressions are shown, *n* = 3. Representative contour plots (values indicate percentage of parent population) and counts of live FcR-blocked **(I,J)** CD45.2^+^CD3^−^CD4^−^CD8^−^NK1.1^+^NKp46^+^ cells and (**K,L)** CD45.2^+^Lin (CD11b, CD11c, CD19, B220, CD49b, CD105, MHC-II, and Ter119)^−^CD3^−^CD4^−^CD8^−^NK1.1^+^CD122^+^ cells in the spleens of WT and *Cd47*^−/−^ littermate mice are shown. Data are representative of two experiments involving 3–4 mice per experiment (Mean ± SEM).

CD47 is ubiquitously expressed, but transcript data in BioGPS (http://biogps.org/#goto=genereport&id=16423) and protein levels detected by flow cytometry indicated the highest expression of CD47 in NK cells among lymphocytes (Figures S1A–C). An antisense morpholino that hybridizes with the 5′-UTR of CD47 mRNA but not a mismatched control morpholino has been documented to lower CD47 expression and functional activity *in vitro* and in various tissues of mice including spleen following IP injection in buffered saline (35, 41). Based on the previously observed weak agonist activity of the mismatched control morpholino, injection of the PBS vehicle was used as control (42). We examined the effect of CD47 blockade on spleen cell homeostasis 14 days after injection (Figure 1A). Functional knockdown of CD47 in hematopoietic cells by the morpholino was validated by the enlarged spleens of CD47 morpholino-treated mice compared to controls (Figure 1B), which is consistent with the increased splenic clearance of red cells and decreased CD47 expression (43). Although, *Cd47*^−/−^ mice exhibit a compensatory down regulation of CD4^+^ DCs that limits red cell clearance (44), naïve *Cd47*^−/−^ mouse spleens exhibited similar enlargement as that observed following morpholino knockdown (Figure 1E). Following CD47-morpholino treatment of WT mice no differences in the splenic CD4 and CD8 T cell compartments were evident by flow cytometric staining, but the CD4^−^CD8^−^CD3^−^ population was significantly increased (Figures 1C,D). Naïve *Cd47*^−/−^ mice exhibited a similar increased CD4^−^CD8^−^CD3^−^ cell population within negatively selected pan T cells from *Cd47*^−/−^ spleens (Figure 1F). ImageStream analysis suggested that these are hematopoietic cells, having comparable cell and nuclear sizes to the CD4 and CD8 T cells (Figure 1G, Figures S2A–C). A lineage-specific gene expression analysis of the sorted CD4^−^CD8^−^CD3^−^ population from isolated pan T cells of *Cd47*^−/−^ mice revealed high mRNA expression of granzyme *B* and perforin. The pan T cell isolation kit from Miltenyi Biotec includes antibodies to deplete both mature (CD11b^+^CD49b^+^) and a subset of immature (B220^+^) NK cells (see material and methods) from mouse splenocytes. However, the sorted CD4^−^CD8^−^CD3^−^ cells from isolated pan T cells had low expression of *Cd3e* (CD3ε), *Tcf7* (TFC-1), *Gata3* (GATA3) and *Rorc* (RORγt) with a concomitant upregulation of *Eomes* (Eomesodermin), *Klrb1c* (NK1.1) and *Ncr1* (NKp46) expression, suggesting these cells to be a subset of immature cells belonging to the NK cell lineage (Figure 1H). Henceforth, the cells obtained by negative selection will be referred to as pan T/iNK cells. The aryl hydrocarbon receptor (*Ahr*), which regulates NK cell cytotoxicity (45), was also highly expressed in this cell population (Figures S3A,B).

Flow cytometric cell surface staining revealed a significant increase in the NK1.1^+^ NKp46^+^ compartment within the CD4^−^CD8^−^CD3^−^ population of *Cd47*^−/−^ spleen, validating the mRNA gene expression data (Figures 1I,J). The Lin^−^CD4^−^CD8^−^CD3^−^ population from *Cd47*^−/−^ spleen expressed significantly more CD122 (IL-2Rβ) receptor, a characteristic of DX5^−^ immature NK (iNK) cells (37), than WT (Figures 1K,L and Figure S4G). In fact, NK1.1^+^NKp46^+^ cells were substantially more prevalent (~1.2 to 3 × 10^6^) than NK1.1^+^CD122^+^ cells (1–2.1 × 10^6^) in spleens of *Cd47*^−/−^ mice, indicating enhanced proliferation and/or differentiation of iNK cells to mNK cells in these mice than in WT mice. Similarly, Lin^−^Eomes^hi^ NK1.1^+^NKp46^+^ and NK1.1^+^CD122^+^ populations were increased in spleen and mesenteric lymph nodes (mLN) of *Cd47*^−/−^ mice (Figures S4A–F).

### CD47 Dose-Dependently Limits Mature NK Cell Numbers in Spleen

**Figure 2:**
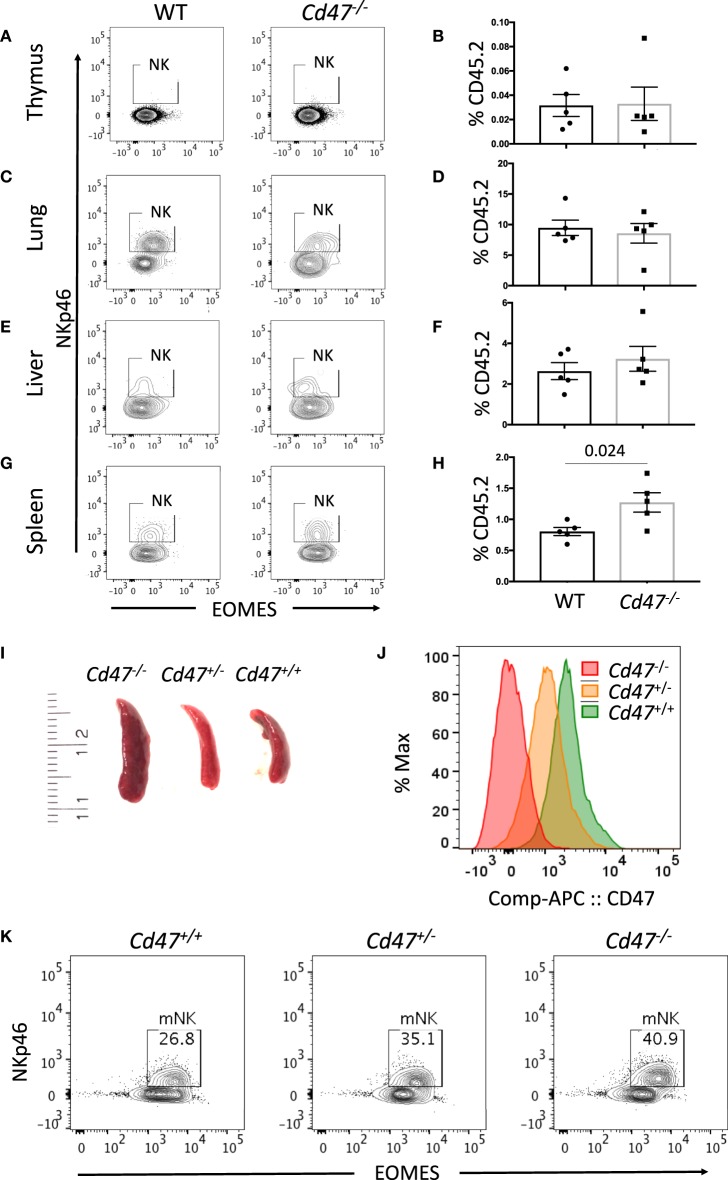
Mature NK cell number varies in mouse spleen in a CD47 dose-dependent manner. Single cell suspensions from thymus, lung, liver and spleen of WT and *Cd47*^−/−^ littermate and sex-matched mice were FcR-blocked and stained for UV Zombie, CD45.2, Lin (B220, CD19, Gr1 and Ter119), NK1.1, and NKp46. Cells were fixed, permeabilized and intracellularly stained for Eomes. **(A–H)** Representative contour plots (values indicate percentage of parent population) and frequency of NKp46^+^ cells which are gated from live, CD45.2^+^Lin^−^NK1.1^+^ cells in the indicated organs of WT vs. *Cd47*^−/−^ mice are shown (*n* = 4). **(I)** Morphologies of spleens from *Cd47*^+/+^, *Cd47*^+/−^, and *Cd47*^−/−^ littermate mice are illustrated. (**J)** Histograms showing the CD47 expression in bone marrow cells of *Cd47*^+/+^, *Cd47*^+/−^, and *Cd47*^−/−^ littermate mice. **(K)** Representative contour plots (values indicate percentage of parent population) showing the CD47-dose dependent change of live mNK (CD45.2^+^Lin^−^NK1.1^+^NKp46^+^Eomes^+^) cells. Data were derived from two experiments involving four to five mice per experiment (Mean ± SEM).

The increased abundance of a subset of iNK cells within the secondary lymphoid organs of CD47-deficient mice prompted us to compare the homeostatic distribution of NK cells in different organs and tissues. NK cells (including both mNK and iNK cells) were rare in thymus but abundant in lung and liver (Figures 2A–F). The frequency of NK cells was significantly increased in spleens from *Cd47*^−/−^ vs. WT mice (Figures 2G,H). Cells from spleens of WT, *Cd47*^+/−^, and *Cd47*^−/−^ littermate mice showed a gradient of cell surface CD47 protein expression, which inversely correlated with spleen size and mNK cell frequencies within the spleens (Figures 2I–K).

### Increased *Cd47^−/−^* NK Cell Proliferation and Associated mNK Numbers in *Cd47^−/−^* Mice

**Figure 3:**
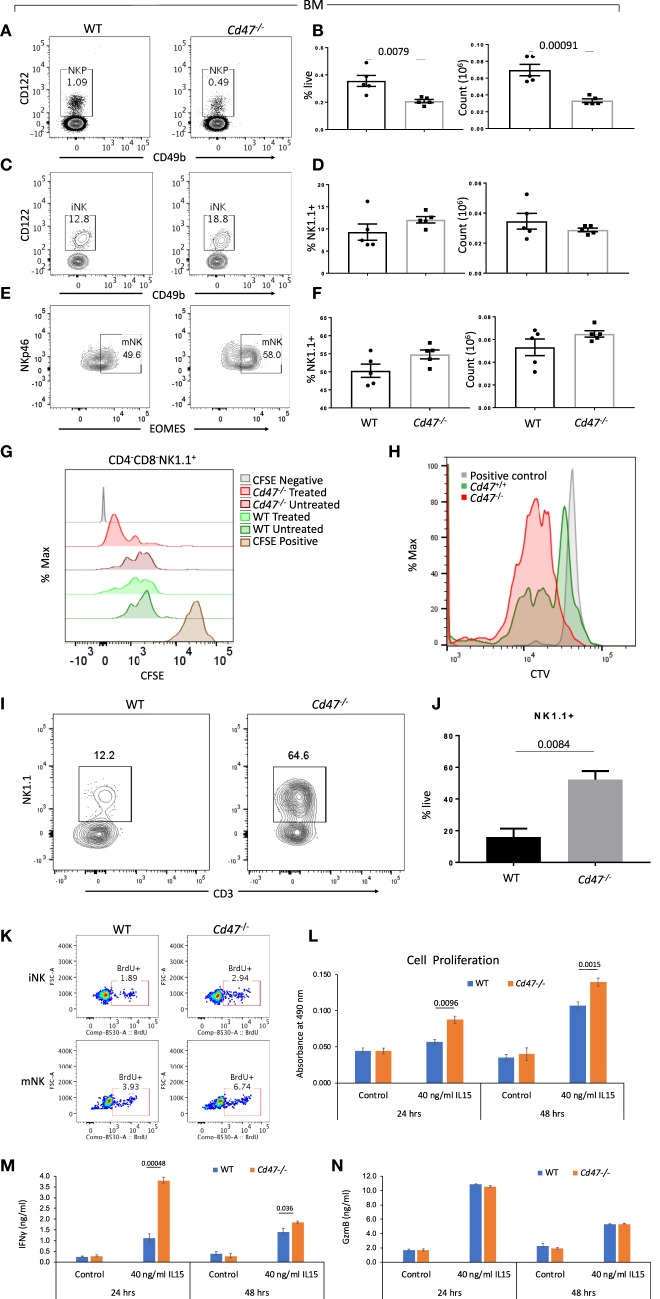
NKP cells in the BM of *Cd47*^−/−^ mice are reduced with a concomitant increase of iNK and mNK cells. Bone marrow (BM) single cell suspensions from WT and littermate *Cd47*^−/−^ mice were stained for Aqua live/dead, Lin (CD3, CD4, CD8, B220, CD19, CD11c, Gr1, and Ter119), CD127, CD122, CD49b, NK1.1, and NKp46. Cells were then fixed, permeabilized and intracellularly stained for Eomes. **(A,B)** Representative contour plots (values indicate percentage of parent population), frequency and count of NK cell precursors (NKP, gated on Lin^−^NK1.1^−^CD49b^−^CD122^+^ cells) **(C,D)** immature NK cells (iNK, gated on Lin^−^CD127^−^NK1.1^+^CD49b^−^CD122^+^ cells) and **(E,F)** mature NK cells (mNK, gated on Lin^−^NK1.1^+^NKp46^+^Eomes^+^ cells) are shown, *n* = 5. **(G)** Splenocytes from WT and *Cd47*^−/−^ littermate mice were enriched for Lin (B220, CD19, CD11b, CD11c, CD49b, CD105, MHC-II, and Ter119)-depleted cells, pulsed with CFSE and cultured on uncoated or anti-CD3 (2 μg/ml) plus anti-CD28 (1 μg/ml) coated plates for 48 h in complete RPMI (10% FCS), CFSE dilutions were measured for cultured CD4^−^CD8^−^NK1.1^+^ cells. (**H)** CTV dilutions are depicted from Lin (B220, CD19, CD11b, CD11c, CD49b, CD105, MHC-II and Ter119)^−^CD4^−^CD8^−^ cells which were sorted from the spleens of WT and *Cd47*^−/−^ mice and cultured for 1 week in complete RPMI + 60 IU/ml IL-2. **(I,J)** Representative contour plots (values indicate percentage of live cells) and frequency of NK1.1^+^ cells within a week in culture were shown, n=3. Data derived are representative of two experiments involving three mice per experiment (Mean ± SEM). **(K)** Incorporation of BrdU in BM iNK and mNK cells of WT and *Cd47*^−/−^ mice, 3 h after BrdU i.p. injection. **(L)** Unlabeled NK cells were isolated from spleens of WT and littermate *Cd47*^−/−^ mice and cultured with/without IL15. Cell proliferation was estimated for 24 and 48 hours using a tetrazolium [3-(4,5-dimethylthiazol-2-yl)-5-(3-carboxymethoxyphenyl)-2-(4-sulfophenyl)-2H-tetrazolium (MTS) proliferation assay. **(M,N)** levels of the secreted cytokine IFNγ and granzyme B (GzmB) in the NK cell culture media were quantified using ELISA kits.

NK cells develop in bone marrow (BM) from the common lymphoid progenitors as a distinct NK cell precursor (NKP) lineage: Lin^−^NK1.1^−^CD49b^−^CD122^+^ (Lin cocktail includes anti-CD3, CD4, CD8, B220, CD19, CD11c, Gr1, and Ter119 antibodies). NKP further differentiate into immature NK cells (iNK: Lin^−^CD127^−^NK1.1^+^CD49b^−^CD122^+^) and mature NK cells (mNK: Lin^−^NK1.1^+^NKp46^+^Eomes^+^) in BM and spleen. Comparing the homeostatic distribution of NKP, iNK and mNK cells in BM and spleen of WT and *Cd47*^−/−^ littermate mice, we observed significant decreases in the frequency and count of NKP cells in BM and spleen of *Cd47*^−/−^ compared to WT mice (Figures 3A,B and Figures S5A–F). Depletion of NKP cells in BM and spleen was associated with a concomitant increase of iNK and mNK cells in the BM and spleens of *Cd47*^−/−^ mice (Figures 3A–F and Figures S5A–F).

To examine effects of CD47 on NK cell homeostatic distribution we performed a mixed bone marrow chimera experiment transferring mixed *Cd47*^−/−^ and WT T- and NK-depleted donor bone marrow cells to irradiated CD45.1 mice. However, consistent with the antiphagocytic function of CD47, *Cd47*^−/−^ donor-derived peripheral NK cells together with other cell lineages were completely eliminated within 8 weeks of transfer (Figures S8A–C).

Independent of increased phagocytic clearance, the deficit of NKP and concomitant increase of mNK in the *Cd47*^−/−^ mice could result from faster proliferation and differentiation of *Cd47*^−/−^ NKP. To test this hypothesis, isolated negatively selected pan T/iNK cells from the spleens of WT and *Cd47*^−/−^ mice were pulsed with CFSE and cultured on anti-CD3 coated plates. As a majority of the isolated cells were T cells, anti-CD3 stimulation would lead to T cell activation-dependent release of cytokines, including IL-2, that promote expansion of the NK lineage cells. After 48 h in culture, NK1.1^+^ cells from *Cd47*^−/−^ mice exhibited increased dilution of CFSE compared to similarly treated WT NK1.1^+^ cells (Figure 3G). To confirm this observation, flow sorted iNK cells (CD4^−^CD8^−^CD3^−^ cells from the isolated pan T/iNK cells, which were also negative for CD11b, CD11c, Dx5, and other lineage markers, from WT and *Cd47*^−/−^ mouse spleens were pulsed with CellTrace violet (CTV) and cultured with IL-2 (60 IU/ml, Figure 3H). The sorted iNK cells from *Cd47*^−/−^ mice diluted CTV more than the WT cells after a week in culture. Moreover, *Cd47*^−/−^ cells differentiated significantly more to a NK1.1^+^ lineage, but none differentiated to a CD3^+^ lineage (Figures 3H–J). We then injected BrdU into WT and *Cd47*^−/−^ littermate mice and 3 h later checked for BrdU incorporation in NK lineage cells within bone marrow. Frequencies of BrdU positive iNK and mNK cells were more in *Cd47*^−/−^ compared to WT mice (Figure 3K).

To directly assess the cell-intrinsic role of CD47 in NK cell proliferation, we cultured isolated NK cells from spleens of WT and littermate *Cd47*^−/−^ mice with IL-15 (40 ng/ml) for 24 and 48 h and assessed proliferation using the MTS assay. Cell proliferation in response to IL-15 was significantly increased in *Cd47*^−/−^ NK cells compared to WT counterparts at both time points (Figure 3L).

To assess the CD47-dependence of functional activation by IL-15, we analyzed the secreted effector proteins IFN gamma and granzyme B in the culture medium of cells treated as above. IL-15 stimulated secretion of both proteins at both time points. A significant increase in IFN gamma secretion was evident in the culture medium of *Cd47*^−/−^ NK cells compared to WT NK cells. In contrast, no differences in granzyme B levels were observed (Figures 3M,N). The increased proliferative and interferon signature in response to IL-15 treatment could also be attributed to the higher abundance of CD122-expressing NK cells in *Cd47*^−/−^ mice than in WT NK cells (Figure S4G).

### CD47 Regulates Global Gene Expression in NK Cells

**Figure 4:**
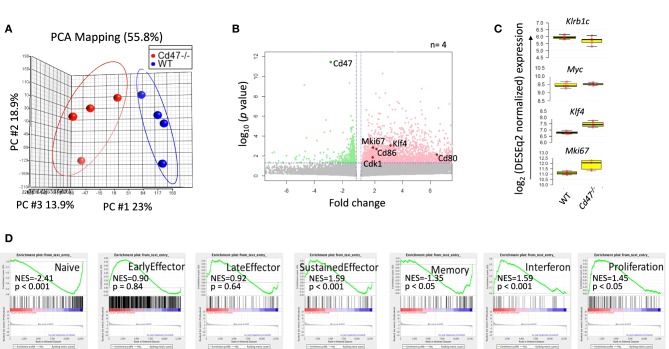
CD47 regulates global gene expression in naive NK cells. Lin^−^NK1.1^+^NKp46^+^ cells were sorted from spleens of WT and *Cd47*^−/−^ mice. Total RNA was isolated from sorted cells, fragmented, cDNA library was prepared followed by paired end sequencing. **(A)** PCA comparing global gene expression between WT and *Cd47*^−/−^ naïve NK cells. **(B)** Volcano plot shows that many genes are significantly upregulated in naïve *Cd47*^−/−^ compared to naïve WT NK cells. **(C)** Boxplots show log2 expression of *Klrb1c, Myc, Klf4*, and *Mki67* in WT and *Cd47*^−/−^ naïve NK cells. **(D)** GSEA plots showing enrichment of early effector, late effector, sustained effector, interferon, memory and proliferation signature genes in naïve *Cd47*^−/−^ over WT NK cells. Data were derived from an experiment involving four mice per group.

CD47 regulates expression of stem cell transcription factors including c-Myc, Sox2, Klf4, and Oct4 in endothelial cells, T cells and various tissues in mice (14). c-Myc is a universal amplifier of expressed genes in lymphocytes, stem cells and tumor cells, and c-Myc regulates several CD47-dependent metabolic programs in Jurkat T cells that are consistent with changes we observed in NK cells (46–48). To assess the role of CD47 in regulating global gene expression in NK cells, we performed RNAseq analysis of WT and *Cd47*^−/−^ NK cells (FACS-sorted Lin-NK1.1^+^NKp46^+^) isolated from naïve mouse spleens. Principal component analysis (PCA) of the WT and *Cd47*^−/−^ NK cell data showed basal differences in gene expression comparing WT and *Cd47*^−/−^ NK cells (Figure 4A). Basal expression of 2,584 genes was significantly increased in *Cd47*^−/−^ NK cells compared to WT NK cells (Figure 4B and Table S1). As expected, *Cd47* was significantly downregulated in *Cd47*^−/−^ NK cells. No significant increase in *c-Myc* was observed, but *Klf4* mRNA expression, which supports maintenance of mNK in spleen (49), was increased 2.6 fold (*p* < 0.001), which correlated with the 1.9-fold increase in *Mki67* (encoding Ki-67, *p* = 0.001) in *Cd47*^−/−^ NK cells (Figure 4C). RNA expression of Itgam (a marker for mNK) and Il2rb (a marker for iNK) did not show any difference between the sorted WT and *Cd47*^−/−^ populations (Figure S7C). These data suggest that the gene expression changes we report do not result from different mNK/iNK ratios in the sorted WT and *Cd47*^−/−^ cells that were used for the RNAseq. Analysis.

Gene set enrichment analysis (GSEA) showed significant negative enrichments for previously reported naïve (NES = −2.41, *p* < 0.001) and memory (NES = −1.35, *p* < 0.05) phenotype NK cell signature genes (50), but a significant positive enrichment of sustained NK effector (NES = 1.59, *p* < 0.001) and interferon (NES = 1.59, *p* < 0.001) signature genes (Qiagen GeneGlobe: Interferon Signaling, species = mouse) in CD47-deficient NK cells (Figure 4D and Table S2). Cell cycle and proliferation signature genes exhibited a significant positive enrichment (NES = 1.45, *p* < 0.05; Qiagen GeneGlobe: Cell Cycle, species = mouse) in *Cd47*^−/−^ NK cells, validating the enhanced proliferative phenotype in these cells (Figure 4D and Table S2).

### CD47-Deficiency Impairs the NK Cell Immune Response to LCMV Infection

NK cells play an essential role in antiviral immunity. The first demonstration of NK cell cytotoxicity during viral infections was in the context of LCMV infection in mice (51). Therefore, the observation that CD47 blockade or genetic deficiency increases the frequency of mature and immature NK cells in spleens of mice prompted us to test the CD47-dependence for LCMV infection. We infected WT and littermate *Cd47*^−/−^ mice with acute Armstrong strain of LCMV. Viral titer analysis in serum on day 3 of acute infection did not show any difference between WT and *Cd47*^−/−^ mice (Figures S6A–B). However, flow cytometric analysis of LCMV Armstrong infected mice at day 8 post infection revealed a significant decrease in the frequency of NK1.1^+^ and NKp46^+^ populations in spleens of *Cd47*^−/−^ compared to WT mice (Figures S6C–F). NK cells from *Cd47*^−/−^ spleen had significantly lower intracellular Eomes levels but comparable T-box transcription factor (T-bet) levels (Figures S6G–I). A significant down-regulation of CD62L and a concomitant upregulation of CD44 were also evident on NK cells from infected *Cd47*^−/−^ mice (Figures S6J–K). Moreover, a tendency to express more PD-1 and decreasing trend of granzyme B and Ki-67 were evident in NK cells of LCMV Armstrong infected *Cd47*^−/−^ mice (Figures S6L–N). These data indicate that *Cd47*^−/−^ NK cells have impaired proliferation and activation responses to acute LCMV infection.

**Figure 5:**
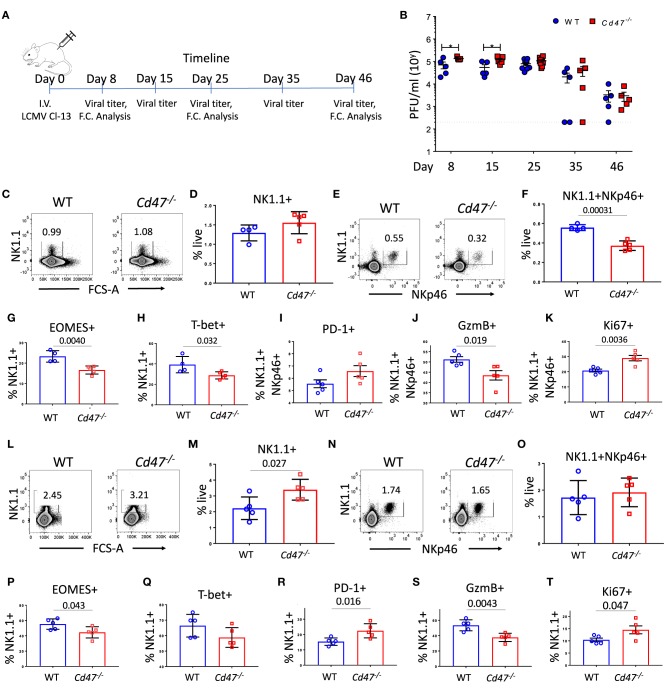
CD47 limits LCMV infection in mice. **(A)** WT and *Cd47*^−/−^ age and sex-matched mice were infected with LCMV Cl-13 (i.v. injection of 2 × 10^6^ pfu/mouse), and timeline shows infection and analysis. (**B**) Viral titers were quantified in serum of LCMV Cl-13 infected mice on the indicated days (D) post infection. On day 25 and day 46 of LCMV Cl-13 infection, mice were euthanized, and single cell suspension of spleens were stained for Aqua live/dead, Lin (CD4, -CD8, -TCRβ, -B220, -Gr1, and -Ter119), NK1.1, NKp46, and PD-1. Cells were then fixed, permeabilized and intracellularly stained for Eomes, T-bet, granzyme B (GzmB) and Ki-67. **(C–F)** Representative contour plots (values indicate percentage of live cells) and frequency of Lin^−^NK1.1^+^ cells and Lin^−^NK1.1^+^NKp46^+^ cells within the spleens on day 25 post infection are shown; **(G–K)** Frequencies of Eomes, T-bet, PD-1, GzmB and Ki-67 positive NK cells are plotted. **(L–O)** Representative contour plots (values indicate percentage of live cells) and frequency of Lin^−^NK1.1^+^ cells and Lin^−^NK1.1^+^NKp46^+^ cells are shown within the spleens on day 46 post infection and **(P–T)** Frequencies of Eomes, T-bet, PD-1, GzmB and Ki-67 positive NK cells *n* = 5. Data obtained from representative of two experiments involving 4–5 mice per experiment **(C–T)**, and more than five experiments comprised of four to seven mice **(B)** per group. (Mean ± SEM).

During chronic Cl-13 LCMV infection, *Cd47*^−/−^ mice had significantly higher virus titers in serum than WT mice on days 8 (1.9-fold more in *Cd47*^−/−^ than WT) and 15 (2.2-fold more in *Cd47*^−/−^ than WT), suggesting viral infection progressed relatively faster in *Cd47*^−/−^ mice (Figures 5A,B). Viral titers still trended higher in the *Cd47*^−/−^ mice on day 25 post-infection (1.5-fold more in *Cd47*^−/−^ than WT). Consistent with these viral titers, NK1.1^+^ NKp46^+^ cell numbers in the spleens of *Cd47*^−/−^ mice were significantly decreased on day 25 post-infection (Figures 5C–F). Significant decreases in intracellular Eomes and T-bet levels in the NK compartment were also evident on day 25 in *Cd47*^−/−^ compared to WT mice (Figures 5G,H). NK cells of infected *Cd47*^−/−^ mice also showed an increasing trend in PD-1 expression level, but significantly less granzyme B level (Figures 5I,J). The proliferation marker Ki-67 was significantly elevated in *Cd47*^−/−^ NK cells (Figure 5K). On day 46 post-LCMV Cl-13 infection, viral titers in sera dropped in both strains of mice, indicating that CD47 is not required for resolution (Figure 5B). Subsequent analysis of the spleens of infected mice on day 46 showed that the NK1.1^+^ and NKp46^+^ cell compartments were restored in both WT and *Cd47*^−/−^ mice, and significantly more NK1.1^+^ cells were found in *Cd47*^−/−^ than in WT mice (Figures 5L–O), mirroring the NK cell frequency in uninfected WT and *Cd47*^−/−^ mice. A significant reduction of intracellular Eomes, but not T-bet levels was still evident in *Cd47*^−/−^ NK cells (Figures 5P,Q). Moreover, PD-1 expression was significantly higher, and granzyme B was significantly lower in day 46 infected *Cd47*^−/−^ mice (Figures 5R,S). Consistent with the data from day 25 post infection, intracellular Ki-67 levels were significantly higher in *Cd47*^−/−^ NK cells at day 46 post infection (Figure 5T). Overall, the acute and chronic LCMV infection models show similarly impaired NK cell responses and immune function in *Cd47*^−/−^ mice.

On day 25 of LCMV Cl-13 infection, there was no difference in the splenic NK1.1^+^ populations of WT and *Cd47*^−/−^ mice (Figure 5D). However, the frequency of NK1.1^+^NKp46^+^ cells significantly decreased in *Cd47*^−/−^ mice at this time (Figures 5E,F). These data suggest an increase in the NK1.1^+^NKp46^−^ population in infected *Cd47*^−/−^ mice. An overall increase of NK1.1^+^NKp46^−^ cells in LCMV Cl-13 infected *Cd47*^−/−^ mice was also evident on day 46 post infection with the increase of NK1.1^+^ population (Figure 5M) but no change in the NK1.1^+^NKp46^+^ population (Figure 5O). The role that CD47 plays in the abundance and function of NK1.1^+^NKp46^−^ cells merits further study.

### Distinct Transcriptional Reprogramming of NK Cells Upon Acute LCMV Infection

**Figure 6:**
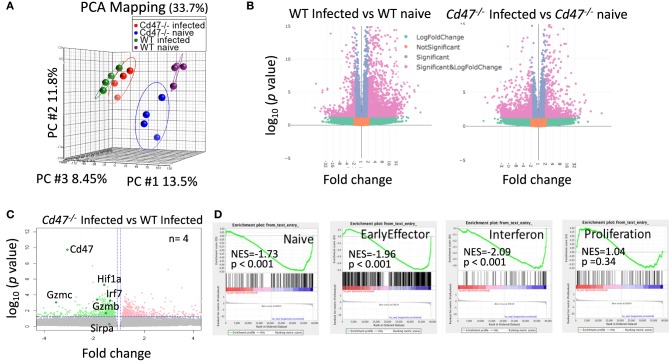
CD47-deficiency limits the transcriptional response during LCMV infection in mice. Lin^−^NK1.1^+^NKp46^+^ cells were sorted from spleens of uninfected and LCMV-infected WT and *Cd47*^−/−^ mice. Total RNA was isolated, fragmented, cDNA library was prepared followed by paired end sequencing. **(A)** PCA on global transcriptome data shows four distinct clusters of naïve WT (purple), naïve *Cd47*^−/−^ (blue), LCMV Armstrong infected WT (green) and LCMV Armstrong infected *Cd47*^−/−^ NK cells (red). **(B)** Volcano plots show broad significant upregulation and downregulation of NK cell gene expression in infected WT and *Cd47*^−/−^ mice compared to their uninfected counterparts. **(C)** Expression pattern of *Cd47, Hif1a, Irf7, Gzmb, Gzmc*, and *Sirpa* are indicated within the volcano plot showing upregulation and downregulation of NK cell gene expression in infected *Cd47*^−/−^ vs. infected WT mice. **(D)** GSEA shows significant negative enrichment of naïve, effector and interferon signature genes in *Cd47*^−/−^ infected compared to WT infected NK cells. Data were derived from an experiment involving four mice per experiment.

**Figure 7:**
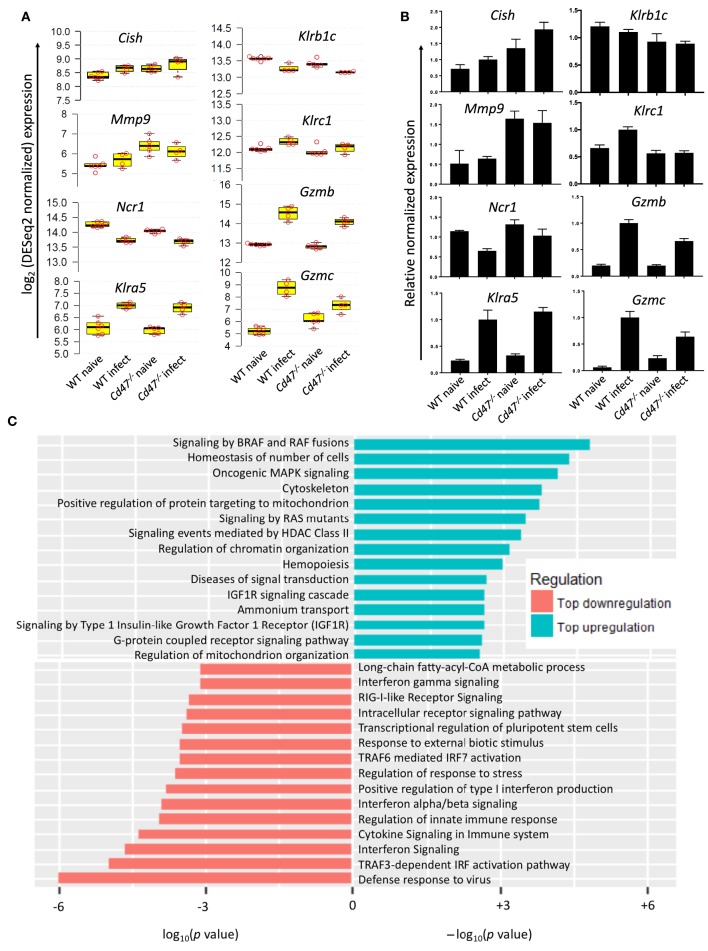
CD47-deficiency impaired NK cell response to virus infection. **(A)** log2 expression of selected genes important for NK cell regulation, suppression and effector function, *n* ≥ 4. **(B)** qRT-PCR validation of the selected genes. **(C)** Waterfall plot shows significant up/down-regulation of distinct pathways in *Cd47*^−/−^ NK cells than in WT NK cells from LCMV Armstrong infected mice.

To identify CD47-dependent gene expression during LCMV infection we infected WT and *Cd47*^−/−^ mice with acute LCMV Armstrong virus and performed global transcriptome analysis of splenic NK cells at day 8-post infection, using naïve mice as controls. Delineation of population relationships with the three most informative principle components showed segregation of the population into discrete clusters (Figure 6A). Comprehensive pairwise comparison of NK cells showed significant upregulation of multiple genes in WT relative to *Cd47*^−/−^ mice upon infection (2,135 genes in WT vs. 486 genes *Cd47*^−/−^ NK cells, Figure 6B, Table S1). Conversely, infection resulted in significant downregulation of more genes in *Cd47*^−/−^ than in WT NK cells compared to the corresponding naïve NK cells (1,136 genes in *Cd47*^−/−^ vs. 447 genes in WT, Figures 6B,C, Table S1). Effector genes including *Hif1a* (HIF-1α), *Irf7* (IRF7), *Gzmb* (granzyme B) and *Gzmc* (granzyme C), together with *Cd47* as a control, were significantly downregulated in *Cd47*^−/−^, less than in WT NK cells upon infection (Figures 6C, 7A,B and Table S1). Comprehensive comparison of GSEA following infection showed significant downregulation of naïve (NES= −1.73, *p* < 0.001), early effector (NES= −1.96, *p* < 0.001) and interferon (NES = −2.09, *p* < 0.001) signature genes in CD47-deficient compared to WT NK cells (Figure 6D, Figure S7A, Table S2). The expressions of *Ncr1* (Nkp46), *Klra5* (Ly-49e), *Klrb1c* (NK1.1), and *Klrc1* (NKG2A) were comparable between NK cells from infected WT and *Cd47*^−/−^ mice. However, the negative regulator *Cish* (CIS) and the suppressor *Mmp9* (MMP9) were significantly upregulated in NK cells of infected *Cd47*^−/−^ mice (Figures 7A,B). Pathway analysis revealed that signaling by BRAF and RAF fusions (R-HSA-6802952), oncogenic MAPK signaling (R-HSA-6802957) and signaling by RAS mutants (R-HSA-6802949) were among the top upregulated pathways in infected *Cd47*^−/−^ NK cells (Figure 7C). The significant downregulation of interferon signature genes, such as *Adar* (DRADA), *Ddx58* (RIG-I), *Ddx60* (DDX60), *Stat1* (STAT1), *Ifih1* (MDA5), *Trim25* (TRIM25), *Irf7* (IRF7), and *Irf9* (IRF9) in infected *Cd47*^−/−^ NK cells is consistent with the impaired *Cd47*^−/−^ NK cell response to viral infection (Figure S7B). Correspondingly, pathway analysis identified the NK cell defense response to viral infection (GO:0051607) as a top downregulated pathway (Figure 7C). Other relevant pathways that were impaired in infected *Cd47*^−/−^ mice included interferon signaling (R-HSA-913531, R-HSA-877300, R-HSA-909733, GO:0032481), immune effector process (GO:0002252), stress response (GO:0080134) and acute inflammatory response (GO:0002526). These gene signatures suggest a global impairment in NK cell effector function in infected *Cd47*^−/−^ compared to WT mice.

## Discussion

NK cells, defined as CD3^−^NK1.1^+^NKp46^+^ in C57BL/6 mice, constitute 5–15% of circulating lymphocytes and are key effectors in anti-viral immunity (52). The present data identify roles for CD47 in murine NK cell homeostasis and function in viral infection that extend beyond the passive role of CD47 as a don't eat me signal. The former passive function of CD47 predicts that knockdown or an absence of CD47 would deplete NK cells, which is consistent with the decreased NKP in *Cd47*^−/−^ BM. But morpholino knockdown of CD47 results in expansion of peripheral NK, and *Cd47*^+/−^ and *Cd47*^−/−^ mice demonstrate a gene dosage-dependent expansion of splenic iNK and mNK cells with decreasing CD47. Our finding is in line with the increased proliferation rate of *Cd47*^−/−^ Foxp3^+^ Tregs (53) and inhibition of T cell proliferation by the CD47 signaling ligand thrombospondin-1 (54, 55). Consistent with those studies, treatment of WT NK cell with thrombospondin-1 reduce intracellular Ki-67 level and proliferation *in vitro* (Nath et al., submitted), suggesting that the accumulation of iNK and mNK cells in *Cd47*^−/−^ mice could be due to the lack of inhibitory thrombospondin-1/CD47 signaling. Consistent with a cell-autonomous inhibitory function of CD47 in NK cells, isolated *Cd47*^−/−^ NK cells exhibited increased IL-15-induced proliferation and IFNγ secretion.

CD47 is expressed at a high level on the surface of NK cells. The immune cell intrinsic role of CD47 is challenging to study *in vivo* using adoptive cell transfer by bone marrow chimera, since deletion of CD47 results in phagocytic elimination of circulating donor *Cd47*^−/−^ cells when transferred to CD45.1 mice (13, 32, 56) (Figures S8A–C). Previous studies showed that expression of CD47 on non-hematopoietic cells regulates the phagocytic activity of macrophages against CD47-deficient hematopoietic cells (57), and macrophages and DCs from CD47 null mice do not phagocytose *Cd47*^−/−^ hematopoietic cells *in vitro* (58). Therefore, *Cd47*^−/−^ mice enable studying the homeostasis of CD47-deficient hematopoietic cells *in vivo*, independent of their phagocytic clearance. CD47-deficient NK cell precursors divide faster *in vitro* and acquire more NK1.1^+^ phenotype than WT counterparts. Therefore, enhanced maturation of NKP cells in *Cd47*^−/−^ mice can account for the reduced frequency of NK cell precursors and concomitant increased iNK and mNK cell frequencies in bone marrow and in spleen.

Our data supports roles for CD47 in NK cell precursor maturation and effector function in response to both acute and chronic viral infection. In contrast to the negative effects of CD47 expression on NK activation and apoptosis, impaired control of melanoma growth in a *Cd47*^−/−^ host implies that CD47 also has positive effects on NK immune functions (Nath et al., submitted). Previous studies have identified cell-intrinsic positive and negative effects of CD47 signaling on T cells homeostasis and effector functions (59), and our data indicates similar negative regulation of NK cells by CD47.

In T cells, CD47 associates with some integrins and promotes their activation, which promotes T cell migration and immune synapse formation. Therefore, defective integrin activation is one potential mechanism to explain the impaired *Cd47*^−/−^ NK responses in LCMV infected mice. CD47-mediated integrin activation is another potential mechanism to account for the decreased NKP abundance in BM of the *Cd47*^−/−^ mice.

Thrombospondin-1 signaling through CD47 is a major inhibitory signaling pathway in T cells and vascular cells (59). Thrombospondin-1/CD47 signaling does not regulate thymic maturation of CD4 and CD8 T cells, but it alters Th1/Th2/Th17 and Treg differentiation (27, 53, 60). Similarly, gene set enrichment indicates that CD47 has distinct effects on naïve, effector, and memory NK cell gene expression. Expanded Th1, Th2, and Th17 populations were found in spleens of mice infected with *Candida albicans* and associated with altered inflammatory gene expression and impaired control of the infection in *Cd47*^−/−^ mouse kidneys (27). Notably, the GeneGo NKG2D signaling pathway was correlated with CD47 expression in spleen of mice infected with *C. albicans* (27). The present data suggest that altered splenic NK cell numbers or activation could account for the latter data and extends the systemic effects of CD47 to splenic NK cells in the context of viral infection. Because CD47 is a ubiquitous regulatory receptor on immune cells, we cannot assign an exclusive role of NK cells in mediating the defect in control of LCMV infection in *Cd47*^−/−^ mice, and targeted deletion of CD47 in immune subsets is not feasible due to its don't eat me function.

Based on the expression of surface receptors, NK cell education/licensing is broadly classified as classical MHC-1 dependent, non-classical MHC-1 dependent, and MHC-1 independent (61). Our RNA-seq data indicates comparable expression of inhibitory *Klra1* and *Klra9* (data not shown) but lower expression of *Klrc1* in *Cd47*^−/−^ than in WT NK cells, which suggest an intact classical but defective non-classical MHC-1 dependent NK arming in *Cd47*^−/−^ animals. The expressions of *Slamf2* and *Smalf6* were also comparable between WT and *Cd47*^−/−^ NK cells after infection (data not shown), also indicating an intact MHC-1 independent NK cell activation. NK cells may also target virus-specific T cells during infection and establish viral chronicity (62, 63). Downregulation of inhibitory *Klrc1* suggests an elevated NK regulatory function in *Cd47*^−/−^ mice (64–66). Future studies will address the mechanism by which CD47 regulates NK cell arming and regulatory functions.

NK cells induce effector function during virus infection and eventually undergo apoptosis, which is an essential mechanism to prevent autoimmunity (67–69). Although T cells showed increased persistence and delayed apoptosis at a site of inflammation in *Cd47*^−/−^ and *Thbs1*^−/−^ mice (70), we found systemic depletion of NK cells during LCMV infection. This suggests that CD47 plays a different role in survival of activated NK cells. The increased mitochondrial proton leak, up-regulation of intracellular ROS accumulation and apoptosis of *Cd47*^−/−^ NK cells under stress (Nath et al., submitted), are potential mechanisms to account for depletion of NK cells in LCMV-infected *Cd47*^−/−^ mice.

Vaccination of *Cd47*^−/−^ mice with influenza virus-like particles induced higher levels of antigen-specific IgG2c isotype dominant antibodies and a greater protection against lethal challenge than in WT mice. The protection mechanism against the viral infection was attributed solely to virus-specific antibodies (71). However, chronic LCMV-infected mice exhibit a severe defect in Fcγ-receptor (FcγR)-mediated antibody effector function (72), which is difficult to reconcile with IgG-mediated protection of *Cd47*^−/−^ mice from LCMV infection. The augmented chronic LCMV infection in *Cd47*^−/−^ mice relative to WT counterparts was associated with reduced numbers of splenic NK cells that exhibited impaired proliferation on day 8 of acute LCMV infection. However, at day 46-post LCMV Cl-13 infection the viral titer in serum was low, as chronicity was established in tissues (73), and splenic NK cells recovered to a level similar to uninfected mice. Ki-67 levels in *Cd47*^−/−^ NK cells on day 46 post-Cl-13 infection were significantly elevated, indicating increased proliferation of *Cd47*^−/−^ NK cells.

Immune cell exhaustion associated with chronic infection is poorly understood (74). Although NK cells are critical effector cells against initiation of infection (52), sustained infection may lead to NK cell exhaustion. Enhanced PD-1, a marker of NK exhaustion and dysfunction was found elevated in CD47-deficient NK cells during LCMV Cl-13 infection similar to that reported in chronic HIV-1 infection (75). A significant reduction of CD62L level in *Cd47*^−/−^ NK cells upon *in vitro* stimulation as well as *in vivo* in LCMV-infected mice indicates reduced functionality of NK cells (76). T-bet and Eomes are the master regulators of NK cell development, maturation and function (77). Both transcription factors were downregulated in exhausted NK cells in leukemic mice (78). Similarly, splenic NK cells from LCMV infected *Cd47*^−/−^ mice exhibited significant reductions of Eomes and T-bet.

In summary, our studies identify important roles of CD47 in regulating NK cell homeostasis, proliferation, and responses to viral infection. The elevation of splenic mNK numbers in CD47-deficient mice or following antisense knockdown of CD47 in WT mice suggests cell intrinsic roles of CD47 on NK cell distinct from the passive role of CD47 as an antiphagocytic marker. Some of the CD47 functions in NK cells defined here, including their increased IL-15-stimulated proliferation and IFNγ production, are consistent with loss of an inhibitory signal mediated by the CD47 signaling ligand thrombospondin-1, but additional CD47 signaling mediated by its lateral interactions with other signaling receptors remain to be investigated in NK cells. These findings have translational implications for the humanized CD47 antibodies and SIRPα decoys currently in clinical trials for cancer patients. Our data indicates that increased susceptibility to viral infections could be a significant side effect of these therapeutics. Additional data to define how those therapeutics impact CD47-SIRPα vs. thrombospondin-1-CD47 signaling in NK cells is required. Given the evidence that NK cells contribute to antitumor and antiviral immunity, potential effects of these experimental therapeutics on human NK cell function in cancer and other immunocompromised patients merit further investigation.

## Data Availability Statement

RNAseq data is deposited on the NCBI Sequence Read Archive (GSE113980). Additional analysis of the sequencing data is available in the Supplementary Files.

## Author Contributions

PR and DR conceived the work and wrote the manuscript. PR designed the study, performed the experiments and analyzed data. PR assisted AG in designing and performing the LCMV infection experiments and data analysis. AG also edited the manuscript. DP-N and JS designed and performed the proliferation, cytokine assays and qRT-PCR analyses. DM assisted PR in ImageStream analysis. MC and AM assisted PR in the RNA-seq analysis, and AM performed all R-based programming. ES critically reviewed the study.

### Conflict of Interest Statement

The authors declare that the research was conducted in the absence of any commercial or financial relationships that could be construed as a potential conflict of interest.

## Abbreviations

Arm: LCMV Armstrong
iNK: immature natural killer cells
LCMV: lymphocytic choriomeningitis virus
mNK: mature natural killer cells
NKG2D: killer cell lectin like receptor K1
NKP: NK cell precursors
SIRPα: signal regulatory protein α.
