# Supplementary material for: CD47 Expression in Natural Killer Cells Regulates Homeostasis and Modulates Immune Response to Lymphocytic Choriomeningitis Virus
Source: Front Immunol. 2018 Dec 20;9:2985. doi: 10.3389/fimmu.2018.02985 (PMC6320676; doi:10.3389/fimmu.2018.02985)
Supplement: Supplementary file 3 [file Table_3.pdf]

table S3. Primers used for qRT-PCR analysis of gene expression.

Gene	Forward primer	Reverse primer
beta-Actin	5'-ATGGAGGGGAATACAGCCC-3'	5'-TTCTTTGCAGCTCCTTCGTT-3'
Ahr	5'- CTCCTTCTTGCAAATCCTGC-3'	5'- GGCCAAGAGCTTCTTTGATG-3'
Cd3e	5'-TCGTCACTGTCTAGAGGGCA-3'	5'-CCTCCTAGCTGTTGGCACTT-3'
Cd3g	5'- TGCCATCCACTTGTACCAAA-3'	5'- ACATGGAGCAGAGGAAGGGT-3'
Cish	5'- CGACTACCTCCGACAGTACC-3'	5'-TGTGACAATCCCTCCTCCAC-3'
cMaf	5'- AAATACGAGAAGCTGGTGAGCAA-3'	5'-CGGGAGAGGAAGGGTTGTC-3'
Eomes	5'-GACCTCCAGGGACAATCTGA-3'	5'-GGCCTACCAAAACACGGATA-3'
Foxp3	5'- CTCGTCTGAAGGCAGAGTCA-3'	5'-TGGCAGAGAGGTATTGAGGG-3'
Gapdh	5'-TGTGTCCGTCGTGGATCTGA-3'	5'-CCTGCTTCACCACCTTCTTGAT-3'
Gata3	5'-AGGATGTCCCTGCTCTCCTT-3'	5'-GCCTGCGGACTCTACCATAA-3'
Gzmb	5'-CATGTAGGGTCGAGAGTGGG-3'	5'-CCTCCTGCTACTGCTGACCT-3'
Gzmc	5'-CAGAGGAGATAATCGGAGGCA-3'	5'- ACGAATTTGTCTCGAACCAGG-3'
Id2	5'- AGAAAAGAAAAAGTCCCCAAATG-3'	5'-GTCCTTGCAGGCATCTGAAT-3'
Ifng	5'-TGAGCTCATTGAATGCTTGG-3'	5'-ACAGCAAGGCGAAAAAGGAT-3'
Il2rb	5'-GACAGGCTCCAGGGAAGAG -3'	5'- CAGGACTGCAGGGAACATCT -3'
Klra5	5'-GATAAAAATATACAACCTCCAGGC-3'	5'- AGCAATGGGCATGGATTG-3'
Klrb1c	5'-CACAGCTGCCATTTTCAGTG-3'	5'-AAGGTTCACATTGCCAGACA-3'
Klrc1	5'- CGAAGGATTCCAGTCCATGA-3'	5'-GGTGTCCTGCATTTCCAAAA-3'
Mmp9	5'- GCAGAGGCATACTTGTACCG-3'	5'- TGATGTTATGATGGTCCCACTTG-3'
Ncr1	5'-GGCTGCTGTTCTCAACACCT-3'	5'-GGCTCACAGAGGGACATACA-3'
Perforin1	5'-TGGAGGTTTTTGTACCAGGC-3'	5'-TAGCCAATTTTGCAGCTGAG-3'

Rorc	5’-TGAGGCCATTCAGTATGTGG-3’	5’-CTTCCATTGCTCCTGCTTTC-3’
Stat3	5’-CTGCTCCAGGTAGCGTGTGT-3’	5’- CTCAGCCCCGGAGACAGT-3’
Stat4	5’- TTGAAGCAGAATTGTTGCCA-3’	5’- CTTTCCTATGGAAATCCGGC-3’
Stat6	5’- CTGGGGTGGTTTCCTCTTG-3’	5’-TGCCCGGTCTCACCTAACTA-3’
Tbx21	5’- GCCAGGGAACCGCTTATATG-3’	5’- GACGATCATCTGGGTCACATTGT-3’
Tcf7	5’-GTGGACTGCTGAAATGTTCG-3’	5’-AGCATCCGCAGCCTCAAC-3’
Tfn	5’- AGGGTCTGGGCCATAGAACT-3’	5’- CCACCACGCTCTTCTGTCTAC-3’
Tox	5’-ACATGTTCTCCCCGTCAAAC -3’	5’- ATTTTATCCTCCTCCAGCCC -3’
Zbtb16	5’- CCCAGTTCTCAAAGGAGGATG-3’	5’- TTCCCACACAGCAGACAGAAG-3’
