# Supplementary material for: CD47 Expression in Natural Killer Cells Regulates Homeostasis and Modulates Immune Response to Lymphocytic Choriomeningitis Virus
Source: Front Immunol. 2018 Dec 20;9:2985. doi: 10.3389/fimmu.2018.02985 (PMC6320676; doi:10.3389/fimmu.2018.02985)
Supplement: Supplementary file 4 [file Data_Sheet_1.docx]

Supplementary Material

CD47 expression in natural killer cell regulates homeostasis and modulates immune response to lymphocytic choriomeningitis virus

Pulak Ranjan Nath^1*^, Arunakumar Gangaplara^2^, Dipasmita Pal-Nath^1^, Ajeet Mandal^3^, Dragan Maric^4^, John M. Sipes^1^, Maggie Cam^3^, Ethan M. Shevach^2^ and David D. Roberts ^1*^

*** Correspondence: Dr. David D. Roberts:** [**droberts@mail.nih.gov**](mailto:droberts@mail.nih.gov)

**Dr. Pulak Ranjan Nath:** [**hellopran2000@gmail.com**](mailto:hellopran2000@gmail.com)

# Supplementary Figures and Table Legends

## Supplementary Figures

**
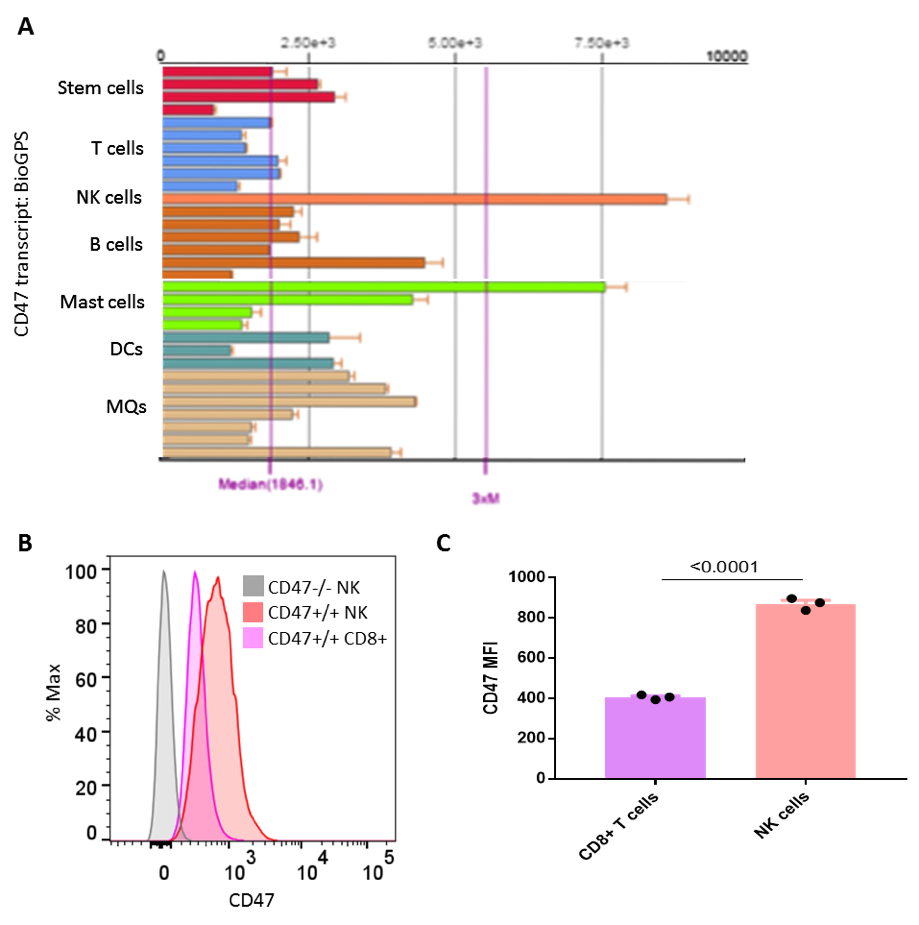
**

**fig. S1. (A)** Bar plot shows CD47 transcript levels in different immune cells in mouse (mm10), acquired from BioGPS portal (<http://biogps.org/#goto=genereport&id=16423>). **(B)** Histograms show the expression of CD47 protein in CD8^+^ T cells and NK cells of C57BL/6 wildtype mice and NK cells from *Cd47*^-/-^ mice (gray), negative control. **(C)** CD47 MFIs were plotted from wild type mice CD8^+^ T cells and NK cells, n= 3. Data derived from representative of two experiments involving three mice per experiment (Mean± SEM).

**
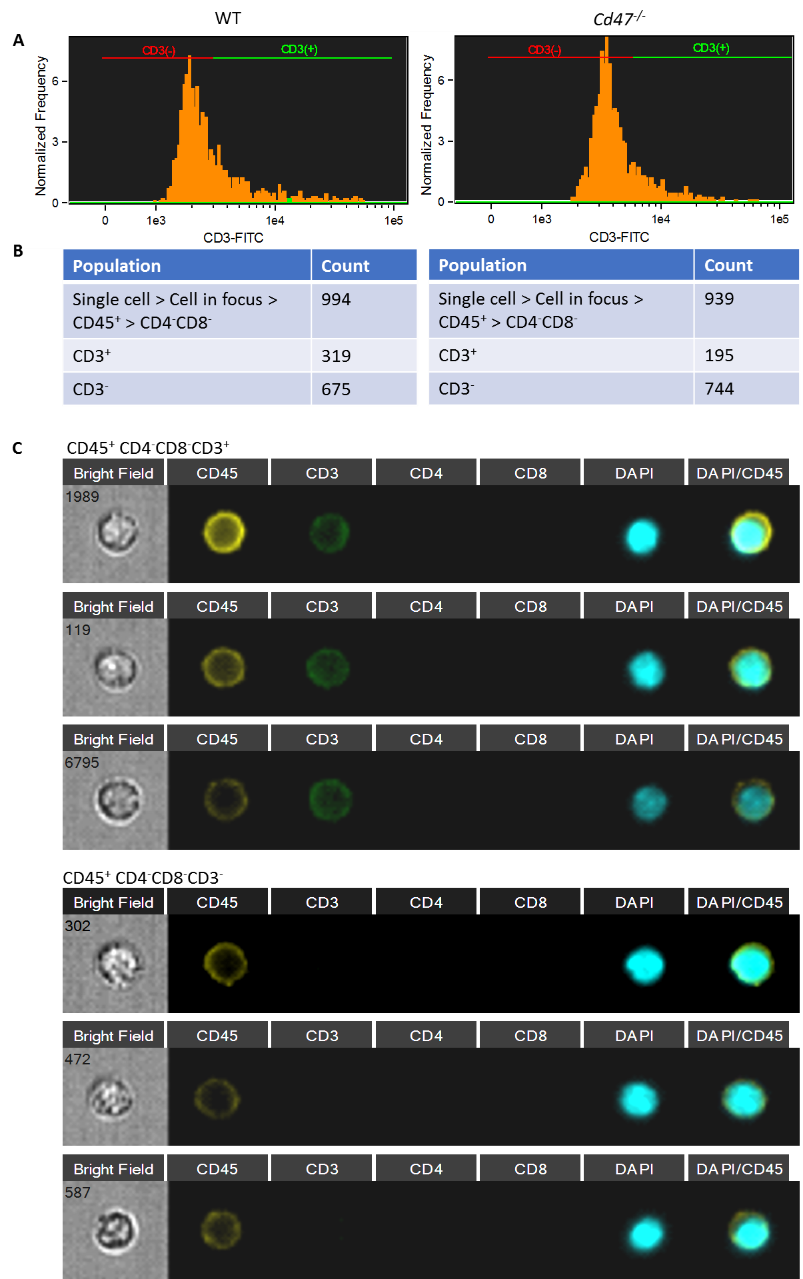
**

**fig. S2. (A)** ImageStream analyzer derived histograms are shown for CD3 expression within the CD4^-^CD8^-^ population of WT and *Cd47*^-/-^ splenic pan T cells after negative enrichment by MACS kit. **(B)** Table shows the counts of CD3^+^ and CD3^-^ subsets within the CD4^-^CD8^-^ population of isolated splenic pan T cells from WT and *Cd47*^-/-^ mice. **(C)** ImageStream analysis shows that splenic CD3^+^ and CD3^-^ subsets of CD4^-^CD8^-^ population from *Cd47*^-/-^ mice are comparable in terms of CD45 expression as well as in cell and nuclear sizes. Representative histograms and ImageStream analysis cells were shown **(A** and **C)**.


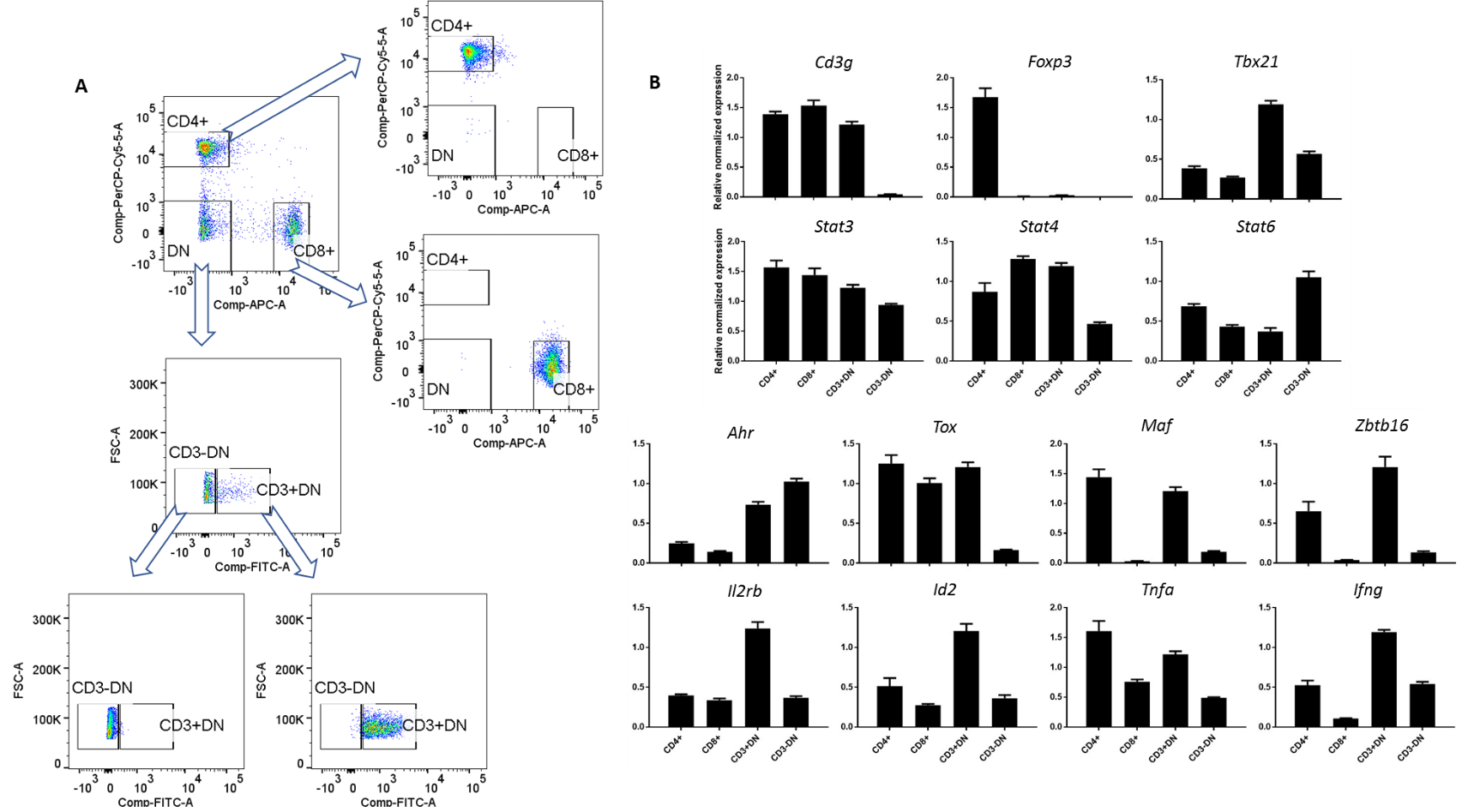


**fig. S3.** Single cell suspensions from spleens of age-matched *Cd47*^-/-^ mice were pooled, and Lin (B220, CD19, CD11b, CD11c, CD49b, CD105, MHC-II and Ter119)-negative cells were enriched using MACS kit. Enriched cells were stained with CD3, CD4 and CD8. **(A)** Representative dot plots showing sorting strategy of indicated populations. **(B)** RT-qPCR expression profile of the indicated genes from sorted CD4^+^, CD8^+^, CD3^+^DN (CD4^-^CD8^-^) and CD3^-^ DN (CD4^-^CD8^-^) cells. *β-Actin* and *Gapdh* are used as internal controls, n=3. Data derived from an experiment involving three mice per group (Mean± SEM).

**
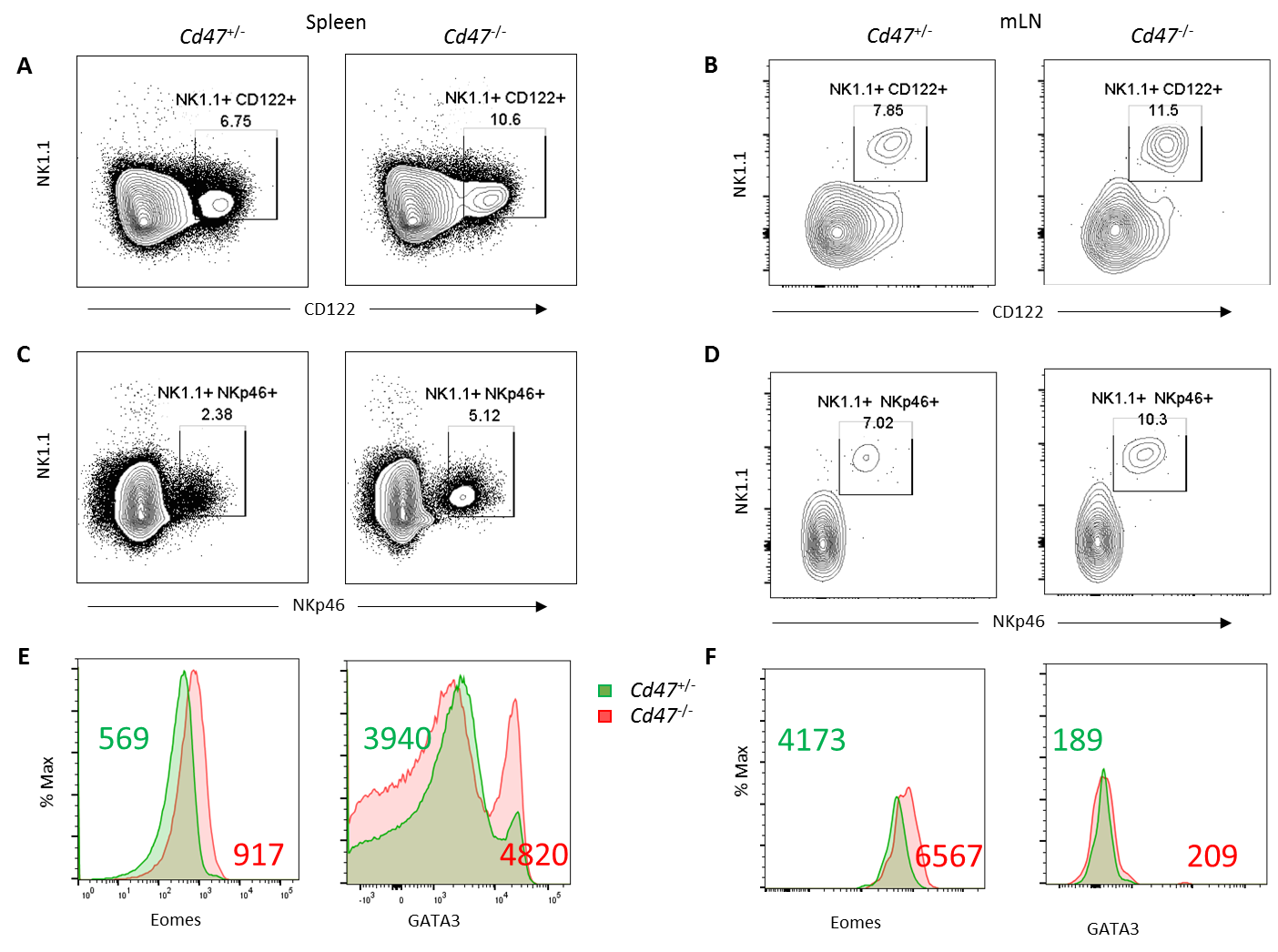
**

G

**fig. S4.** Single cells from spleen and mesenteric lymph node (mLN) of *Cd47*^+/-^ and *Cd47*^-/-^ littermate female mice were FcR-blocked and then stained for CD45.2, Lin (CD11b, CD11c, CD19, B220, Gr1 and Ter119), CD4, CD8, NK1.1, NKp46 and CD122. Cells were then fixed, permeabilized and stained for intracellular Eomes and GATA3. **(A, B)** FACS contour plots show the frequency of live CD45.2^+^Lin-NK1.1^+^CD122^+^ cells in *Cd47*^+/-^ and *Cd47*^-/-^ mouse spleen and mLN. **(C, D)** FACS contour plots show the frequency of live CD45.2^+^Lin-NK1.1^+^NKp46^+^ cells in *Cd47*^+/-^ and *Cd47*^-/-^ mouse spleen and mLN. **(E, F)** Histogram plots show expression of Eomes and GATA3 in CD45.2^+^Lin^-^CD4^-^CD8^-^ cells from spleen and mLN of *Cd47*^+/-^ and *Cd47*^-/-^ mice. **(G)** Histogram plots show expression of CD122 in CD45.2^+^Lin^-^NK1.1^+^ cells from spleen of WT and *Cd47*^-/-^ mice.


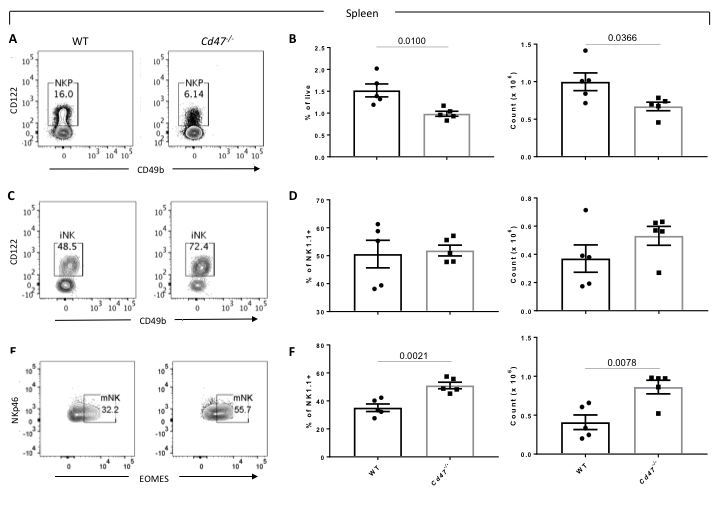


**fig. S5.** Single cell suspension of spleens from C57BL/6 WT and littermate *Cd47*^-/-^ mice were prepared following RBC lysis. Cells were FcR-blocked and stained for Aqua live/dead, Lin (CD3, CD4, CD8, B220, CD19, CD11c, Gr1 and Ter119), CD127, CD122, CD49b, NK1.1 and NKp46. Cells were then fixed, permeabilized and intracellularly stained for Eomes. **(A-B)** Representative contour plots (values indicate percentage of parent population), frequency and count of NKP in spleen of WT and *Cd47*^-/-^ mice were shown. **(C-D)** Representative contour plots (values indicate percentage of parent population), frequency and count of immature NK cells (iNK, Lin^-^CD127^-^NK1.1^+^CD49b^-^CD122^+^) in spleen of WT and *Cd47*^-/-^ mice were shown. **(E-F)** Representative contour plots (values indicate percentage of parent population), frequency and count of mature NK cells (mNK, Lin-NK1.1^+^NKp46^+^Eomes^+^) in spleen of WT and *Cd47*^-/-^ mice were shown, n=5. Data shown representative of two independent experiments involving five mice per experiment (Mean± SEM).

**
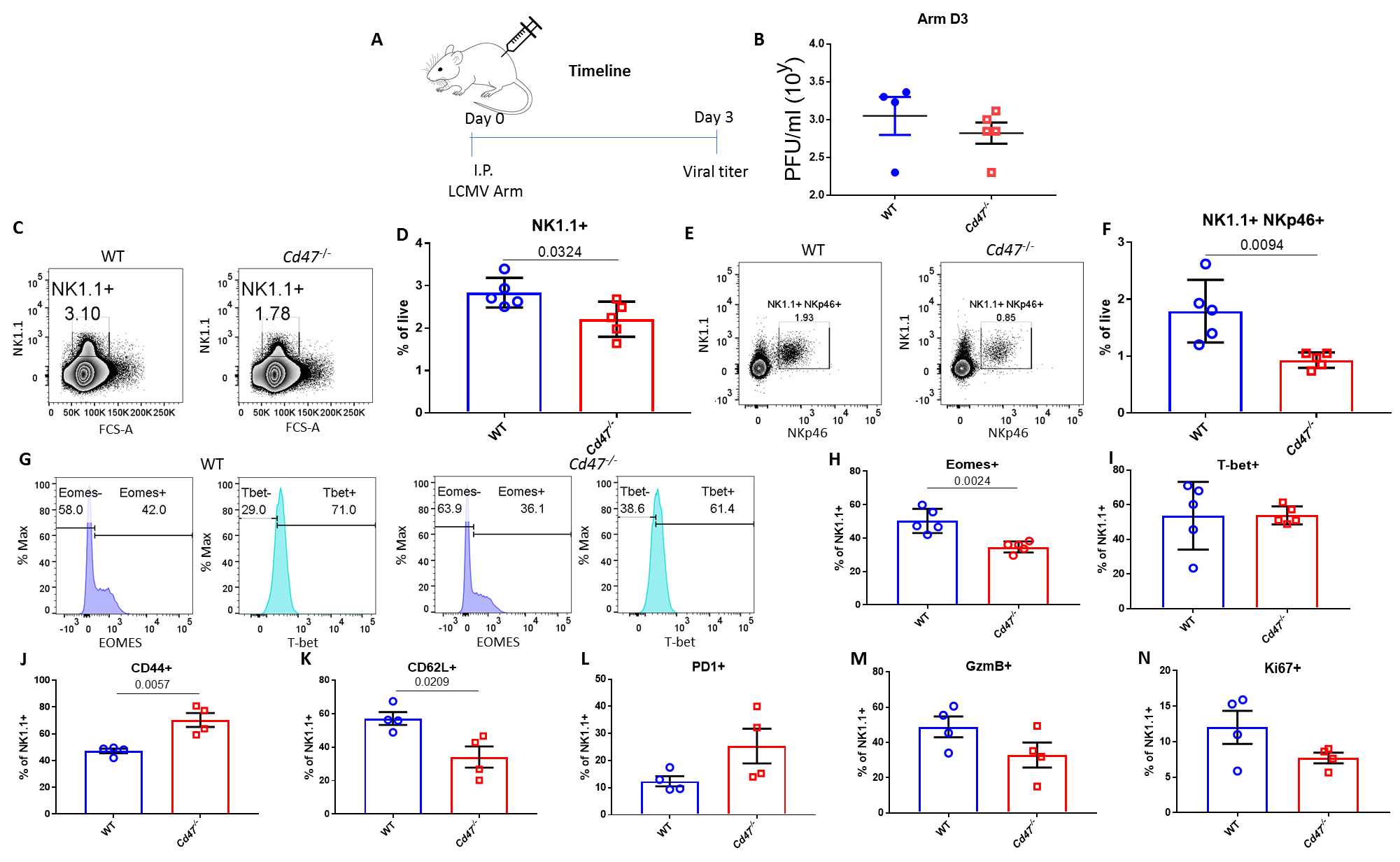
**

**fig. S6.** **(A)** WT and *Cd47*^-/-^ age and sex-matched mice were infected with LCMV Armstrong (i.p. injection of 2x10^5^ pfu/mouse), and timeline shows infection and analysis. **(B)** Sera were collected, and viral titers were quantified in serum of LCMV Armstrong infected mice on day 3 post infection.

On day 8 post infection, mice were euthanized, and single cell suspension of spleens were stained with Lin-cocktail (anti-CD4, -CD8, -TCRβ, -B220, -Gr1 and -Ter119), anti-NK1.1, anti-NKp46, anti-CD44, anti-CD62L and anti-PD-1 in FACS buffer containing Aqua live/dead. Cells were then fixed, permeabilized and intracellularly stained for Eomes, T-bet, granzyme B (GzmB) and Ki-67. Representative contour plots (values indicate percentage of live cells) and frequency of **(C-D)** Lin^-^NK1.1^+^ cells, and **(E-F)** Lin^-^NK1.1^+^NKp46^+^ cells are shown within the spleens on day 8 post infection. **(G-I)** Representative contour plots (values indicate percentage NK1.1^+^ cells) and frequencies of Eomes and T-bet expression in NK cell population of infected WT and *Cd47*^-/-^ mice are shown. **(J-N)** Frequencies of Eomes, T-bet, CD44, CD62L, PD-1, GzmB and Ki-67 positive NK cells within the spleens on day 8 post infection are shown, n=5. Data are representative of two independent experiments involving five mice per group (Mean± SEM).


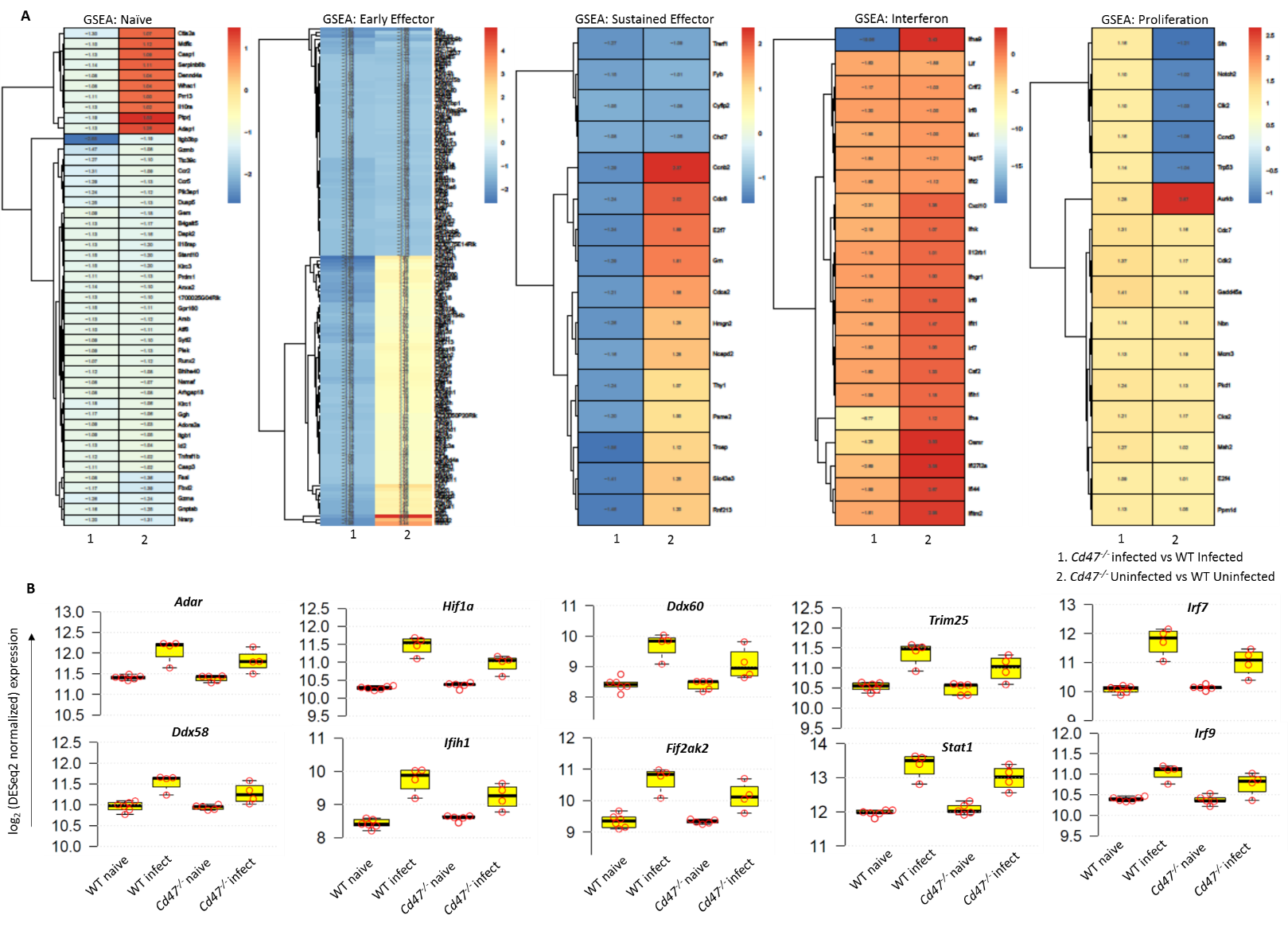
**C**

Log2

Log2

**fig. S7. (A)** Heatmaps show expression pattern of GSEA enriched genes in *Cd47*^-/-^ infected versus WT infected in the first lane and their corresponding expression pattern in *Cd47*^-/-^ uninfected versus WT uninfected in the second lane. **(B)** log2 expression of critical genes in NK cell response to viral infection pathways in NK cells of naïve and LCMV Armstrong infected WT and *Cd47*^-/-^ mice. **(C)** RNA expression of *Itgam* (marker for mNK) and *Il2rb* (marker for iNK) did not show any difference between the sorted naïve WT and cd47-/- populations.


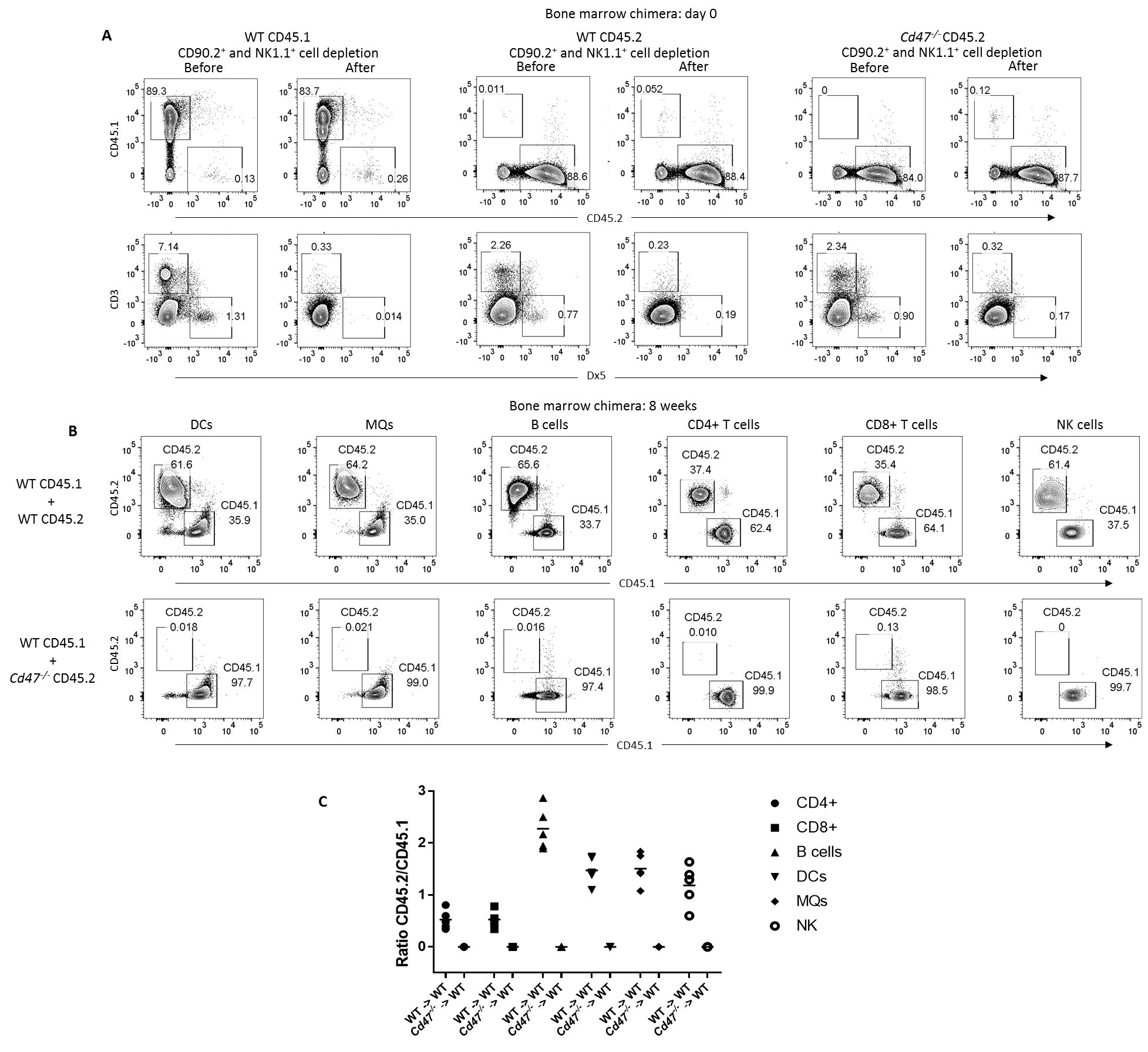


**fig. S8. (A)** Analysis of bone marrow chimera derived cells from CD45.1, WT (CD45.2) and *Cd47*^-/-^ (CD45.2) mice for the expression of CD45.1, CD45.2, CD3, Dx5 and CD47 before and after CD90.2^+^ and NK cell depletion. **(B)** Bone marrow cells reconstitution of CD45.2^+^ and CD45.1^+^ cells and stained DC, MQ, B cell, CD4^+^ T cell, CD8^+^ T cell and NK cells in the peripheral blood of recipient mice after 8 weeks of adoptive bone marrow transfer are shown. **(C)** Graph shows CD45.2 to CD45.1 ratio in the indicated cell compartments in the recipient peripheral blood at 8 weeks post adoptive bone marrow transfer, n=5. Data derived from an experiment involving five mice per group.

## Supplementary Tables

**table S1.** Gene list of DEG normalized and log_2_ transformed expression values of significantly differentially expressed genes within the following contrasts: *Cd47^-/-^* Uninfected vs WT Uninfected, WT LCMV-Armstrong Infected vs WT Uninfected, *Cd47^-/-^* LCMV-Armstrong Infected vs *Cd47^-/-^* Uninfected and *Cd47^-/-^* LCMV-Armstrong Infected vs WT LCMV-Armstrong Infected.

**table S2.** GSEA gene ranklist of NK cell genes from WT and *Cd47^-/-^* uninfected and LCMV-Armstrong infected mice within the following signatures: Naïve, Early Effector, Sustained Effector, Late Effector, Memory, Interferon, Cell Cycle & Proliferation and Apoptosis & Cell Death.

**table S3**. Primers used for qRT-PCR analysis of gene expression.
